# Identifying positive and negative deviants and factors associated with healthy dietary practices among young schoolchildren in Nepal: a mixed methods study

**DOI:** 10.1186/s40795-023-00700-5

**Published:** 2023-03-08

**Authors:** Prasant Vikram Shahi, Rachana Manandhar Shrestha, Pepijn Schreinemachers, Akira Shibanuma, Junko Kiriya, Ken Ing Cherng Ong, Masamine Jimba

**Affiliations:** 1 Department of Community and Global Health, Graduate School of Medicine, The University of Tokyo, 7-3-1 Hongo, Bunkyo-ku, Tokyo, 113-0033 Japan; 2 World Vegetable Center, Bangkok, Thailand

**Keywords:** Dietary diversity score, Nutrition, Qualitative, Quantitative, School and home garden, Nepal

## Abstract

**Background:**

School-based interventions have been implemented in resource-limited settings to promote healthy dietary habits, but their sustainability remains a challenge. This study identified positive deviants (PDs) and negative deviants (NDs) from the control and treatment groups in a nutrition-sensitive agricultural intervention in Nepal to identify factors associated with healthy dietary practices.

**Methods:**

This is an explanatory mixed methods study. Quantitative data come from the endline survey of a cluster randomized controlled trial of a school and home garden intervention in Nepal. Data were analyzed from 332 and 317 schoolchildren (grades 4 and 5) in the control and treatment group, respectively. From the control group, PDs were identified as schoolchildren with a minimum dietary diversity score (DDS) ≥ 4 and coming from low wealth index households. From the treatment group, NDs were identified as schoolchildren with a DDS < 4 and coming from high wealth index households. Logistic regression analyses were conducted to identify factors associated with PDs and NDs. Qualitative data were collected through in-depth phone interviews with nine pairs of parents and schoolchildren in each PD and ND group. Qualitative data were analyzed thematically and integrated with quantitative data in the analysis.

**Results:**

Twenty-three schoolchildren were identified as PDs, and 73 schoolchildren as NDs. Schoolchildren eating more frequently a day (AOR = 2.25; 95% CI:1.07–5.68) and whose parents had a higher agricultural knowledge level (AOR = 1.62; 95% CI:1.11–2.34) were more likely to be PDs. On the other hand, schoolchildren who consumed diverse types of vegetables (AOR = 0.56; 95% CI: 0.38–0.81), whose parents had higher vegetable preference (AOR = 0.72; 95% CI: 0.53–0.97) and bought food more often (AOR = 0.71; 95% CI: 0.56–0.88) were less likely to be NDs. Yet, schoolchildren from households with a grandmother (AOR = 1.98; 95% CI: 1.03–3.81) were more likely to be NDs. Integrated results identified four themes that influenced schoolchildren’s DDS: the availability of diverse food, the involvement of children in meal preparation, parental procedural knowledge, and the grandmother’s presence.

**Conclusion:**

Healthy dietary habit can be promoted among schoolchildren in Nepal by encouraging parents to involve their children in meal preparation and increasing the awareness of family members.

**Supplementary Information:**

The online version contains supplementary material available at 10.1186/s40795-023-00700-5.

## Background

A lack of diet diversity is a major cause of malnutrition [1] in children, thereby affecting their nutritional status [2]. Thus, consuming diverse foods ensures the attainment of balanced nutritional requirements [3, 4]. However, consuming less diverse foods may increase the probability of malnutrition, even leading to cognitive impairment [5].

Cultivating healthy dietary habits during early childhood is crucial [6, 7]. School-based interventions can have a lifelong impact on dietary behavior [8]. School gardens improve children’s knowledge of healthy eating, help identify healthy food, and improve their preference for fruits and vegetables [9–11]. Moreover, home gardens are associated with increased dietary diversity [12, 13].

In Nepal, the World Vegetable Center designed a project entitled, “Nudging children toward healthier food choices: An experiment combining school and home gardens” (hereafter “school and home garden project”). The project taught schoolchildren about gardening and nutrition through a school garden while their parents were trained in home vegetable garden and nutrition [14]. It encouraged healthy eating practices among schoolchildren, providing a positive environment at school and at home. An impact evaluation of the project, using a cluster randomized controlled trial design, showed that parents increased their food and nutrition knowledge, their liking for vegetables and their productivity of their home garden while schoolchildren improved their liking for vegetables and increased their vegetable consumption by 15–26% depending on the season [14].

The project focused on the frequency of vegetable consumption as the main outcome. Most of children already consumed vegetables, and dietary diversity score (DDS) data were therefore thought not to be sensitive enough to measure an increase in vegetable consumption [14]. The DDS of schoolchildren, defined as consuming different food groups [15], could be further improved. The intervention was implemented as a pilot and some schools may have discontinued the school garden after the project ended while others may have continued even without support [16]. Applying locally-available approaches can efficiently bring positive and sustainable changes to the community [17]. There might be positive deviants (PDs) with unusual behavior and strategies in any community, showing outstanding results relative to other community members sharing similar resources and constraints [17–19].

Deviants may also be negative. Negative deviants (NDs) do not imply reciprocal PDs. NDs (individuals or groups) cannot utilize or benefit from a specific program while others in the community do benefit. Factors associated with being NDs help identify non-programmatic components that hinder participants from achieving a positive outcome [20]. Identifying the factors associated with PDs and NDs may help develop an effective and more sustainable program, contributing to children’s nutritional status in resource-limited settings. Thus, this study aimed to (i) identify PDs among those households did not receive support and NDs among those who did receive support from the project, (ii) determine the factors associated with being PDs and NDs, and (iii) identify factors associated with healthy dietary practices.

## Methods

### Study design and study population

This study employed an explanatory mixed methods design. Regarding the quantitative analysis, the study employed secondary data from the endline survey data of the project, collected in June 2019. However, some household and demographic data missing in the endline survey, such as household assets, education, and gender, were extracted from the baseline data (collected in June 2018). Additional file 1 presents the project outline [14].

**Fig. 1:**
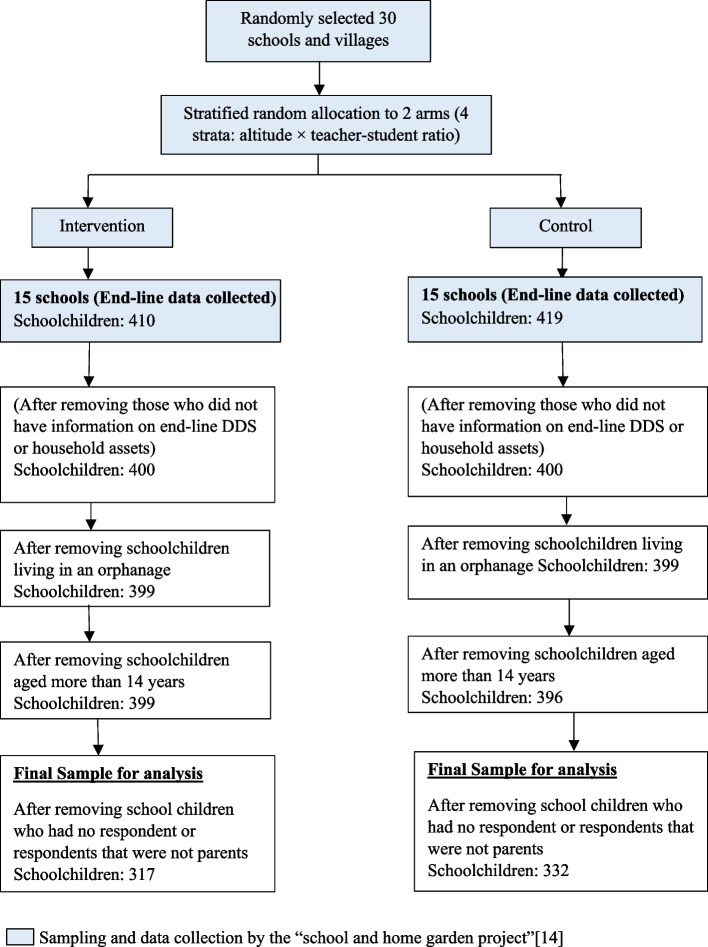
Participant selection flow diagram

The population for this study comprised grade 4 and 5 schoolchildren and their parents from Shindhupalchok, Nepal who were involved in the “school and home garden project”. The exclusion criteria for the analysis were schoolchildren who did not complete the endline food logbook, did not have household asset information, lived in an orphanage, and had no parent respondents. Moreover, three students above 14 years old were excluded from the control group to make groups more homogeneous. The flow diagram (Fig. 1. Participant selection flow diagram) shows the complete participant selection process. Finally, the sample size was 332 and 317 schoolchildren in the control and treatment group, respectively (Fig. 1).

For the qualitative part, primary data were collected through in-depth phone interviews of nine pairs of parents and children in each PD and ND group in October 2020. The exclusion criteria were PD and ND who were ill, whose contact was changed, and refused to participate. Additional file 2 presents the in-depth-interview procedure. Semi-structured, open-ended in-depth interview guidelines were prepared (see additional file 3).

### Variables

**Fig. 2:**
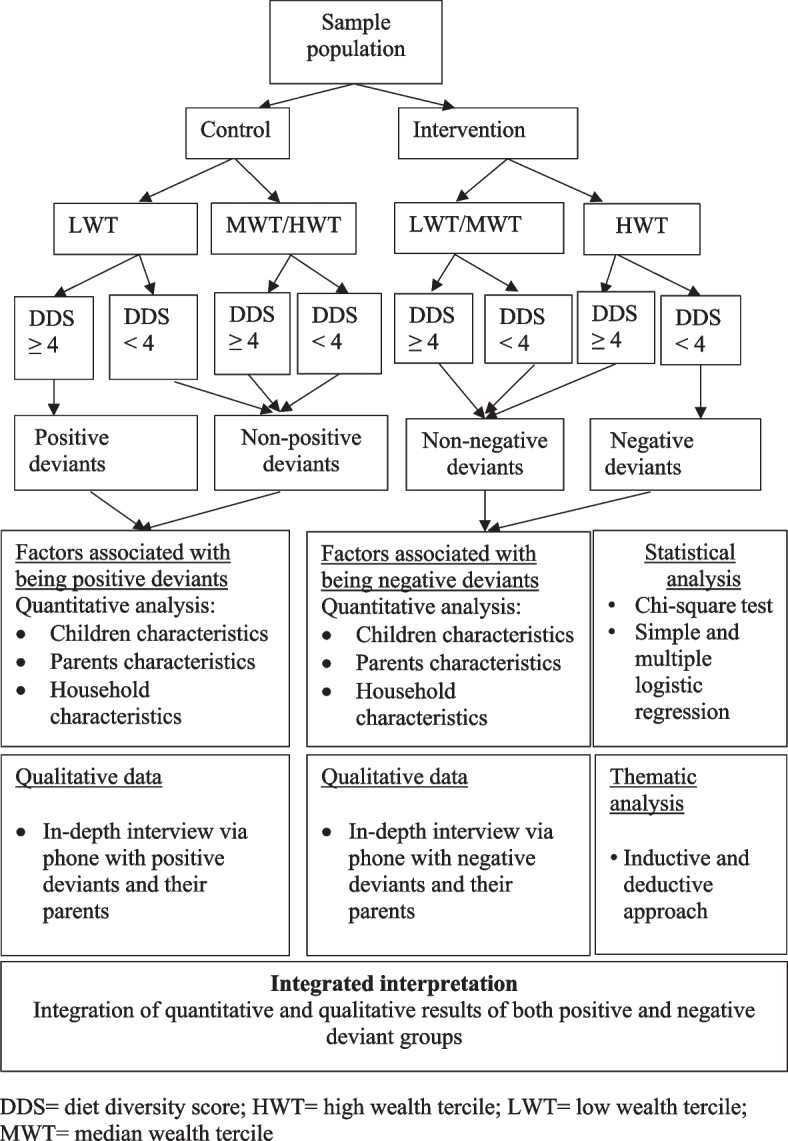
Analysis framework

Being PD or ND was the outcome variable in this study. This was identified based on two criteria: DDS and wealth index (Fig. 2. Analysis framework ). DDS is a proxy for the nutritional adequacy of an individual’s diet [3]. The household wealth index was chosen because socioeconomic factors are positively associated with DDS [21]. PDs (from the control group) were schoolchildren belonging to household with a comparatively low wealth index but nevertheless had, at least, a minimum DDS (≥ 4 DDS). Other children from the same group were defined as non-PDs. However, NDs (from the treatment group) were schoolchildren with a comparatively high wealth index but had nevertheless a lower than the minimum DDS. Other children from the same group were defined as non-NDs (Fig. 2).

Endline DDS was used for analyzing individual DDS. For reliability, we checked the correlation between endline DDS and mean 3-month DDS for each student. The endline DDS correlated strongly (ρ = 0.7) with the 3-month-logbook-mean-DDS. The dataset had 16 food group categories [15]. They were recategorized into seven categories. The minimum DDS was calculated based on the food category consumed by the schoolchildren. The minimum DDS for schoolchildren was the consumption of at least four of seven food categories in a 24-h dietary recall [22]. The seven food groups are (i) grains, roots, and tubers; (ii) legumes and nuts; (iii) dairy products (milk, yogurt, cheese); (iv) eggs; (v) vitamin-A-rich fruits and vegetables; (vi) other fruits and vegetables; and (vii) flesh foods (meat, fish, poultry and liver/organ meats) [22].

The wealth index was calculated using principal component analysis [23] on the household asset variables. The asset variables were recoded into binary variables. After running the analysis, the second factor was selected as it had a high eigenvalue, a minimal difference (0.3) from the first factor’s eigenvalue, and had positive factor loadings in all the asset variables. The score of the second factor was divided into wealth tercile, namely, low, medium, and high. [23].

The independent variables were child characteristics, parent characteristics, household characteristics, and community-level variables. Additional file 1 describes the questionnaire. The preference, knowledge, and practice variables were computed as composite variables from their respective different measures (additional file 1). These composite variables were centered and standardized before treatment in the logistic regression to obtain a standardized logistic regression coefficient [24]. Children’s ethnicity was categorized into three major ethnic groups: Brahaman/Chhetri, Dalits, and Adivasi/Janajatis [25].

### Data analysis

The data analysis comprised two parts: quantitative and qualitative analysis (Fig. 2. Analysis framework ).

#### Quantitative data

Descriptive statistics were used to summarize the characteristics of the schoolchildren, parents, and their households in the control and treatment group. Food consumption was compared between the PDs and non-PDs and NDs and non-NDs. The difference in proportions (food consumed) was measured using the chi-square (χ2) test. Simple and multiple (Firth’s) logistic regression analyses were performed to identify the association between dependent and independent variables. Firth’s (penalized) logistic regression was performed due to the small number of the events (PD and ND) [26–28]. Independent variables for the multiple logistic regression model were selected based on literature review [14, 29, 30]. First, we examined the correlation between continuous variables for multicollinearity. Variables were excluded if the correlation coefficient was higher than 0.7 [31]. The variance inflation factor was less than 2, suggesting that multicollinearity was not a problem [32]. Statistical significance was set at *p* < 0.05. Statistical analysis was performed using RStudio 1.3.1093 (2020) [33] and Stata 13.1 (StataCorp, College Station, TX, United States). This study followed the Strengthening the Reporting of Observational studies in Epidemiology (STROBE) guideline.

#### Qualitative data

The interview recordings were transcribed verbatim by the research assistants. All transcripts were read thoroughly and coded using deductive and inductive approaches. The researcher and the research assistant performed the coding and employed Microsoft Excel for data management, merging similar codes into themes. The consolidated criteria for reporting qualitative research (COREQ) were followed for this study.

Finally, quantitative and qualitative results of both PD and ND groups were compared and interpreted.

## Results

### Quantitative results

In the control group, the mean age of the schoolchildren was 10.4 (standard deviation [SD] 1.5) years, and the mean age of the respondent (parents) was 36.1 (SD 7.0) years. Of the respondents, 87% were mothers. Of the 332 schoolchildren, approximately 23% had the minimum DDS. Based on pre-determined criteria, 23 schoolchildren were PDs. The mean age of PDs and non-PDs were 10.4 (SD 1.5) years and 10.5 (SD 2.1) years, respectively.

The schoolchildren in the treatment group had an average age of 10.3 (SD 1.5) years, while parents’ mean age was 36.3 (SD 7.2) years. Most parents (92%) were mothers. Among the 317 schoolchildren, approximately 26% had a minimum DDS. After applying the criteria, 73 participants were identified as NDs. The mean age of NDs and non-NDs were 10.1 (SD 1.4) years and 10.4 (SD 1.5) years, respectively.

Table 1 shows the characteristics of schoolchildren, parents and household in the control group (stratified by PDs and non-PDs) and in the treatment group (stratified by NDs and non-NDs). For control group, girls made up 56% of the sample schoolchildren. Moreover, approximately 55% were of Adivasi/Janajatis ethnicity, followed by Brahmin/Chhetri (35%) and Dalits. More than half of the parents (56%) could not read or write; most had farming as their major occupation. Most households (87%) produced vegetables in their home gardens with mothers being the primary caretaker of the home garden in 87% of the cases.

**Table 1:** Characteristics of the schoolchildren, parents, and household, in the control group and treatment group (stratified by deviants)^±^

**Variables**	**Category**	**Control group (*n* = 332)**			**Treatment group (*n* = 317)**		
		**Positive Deviant (*n* = 23)** ^*^	**Non-positive Deviant (*n* = 309)**	**Total n (prop.)**	**Negative deviants (*n* = 73)**	**Non-negative Deviants (*n* = 244)**	**Total n (prop.)**
Sex	Boy	0.39	0.44	146 (0.44)	0.48	0.44	143 (0.45)
	Girl	0.61	0.56	186 (0.56)	0.52	0.56	174 (0.55)
Ethnicity	Brahaman/Chhetri	0.17	0.36	117 (0.35)	0.29	0.29	93 (0.29)
	Dalits	0.13	0.10	33 (0.10)	0.04	0.09	24 (0.08)
	Adivasi/Janajatis	0.70	0.54	182 (0.55)	0.67	0.62	200 (0.63)
Parents’ literacy	Illiterate	0.52	0.56	186 (0.56)	0.49	0.61	184 (0.58)
	Literate^†^	0.48	0.44	146 (0.44)	0.51	0.39	133 (0.42)
Parents’ Occupation	Farmer	0.78	0.78	259 (0.78)	0.79	0.83	261 (0.82)
	Not farmer	0.22	0.22	73 (0.22)	0.21	0.17	56 (0.18)
Grandmother in household	Yes	0.26	0.21	72 (0.22)	0.36	0.24	84 (0.27)
Decision-maker—for what to cook	Mother	0.83	0.87	288 (0.87)	0.89	0.91	288 (0.91)
	Other family member	0.17	0.13	44 (0.13)	0.11	0.09	29 (0.09)
Produced vegetables in home garden	Yes	0.78	0.88	289 (0.87)	0.99	0.97	309 (0.97)
Responsible for managing home garden^**‡**^	Mother	0.94	0.87	252 (0.87)	0.86	0.87	269 (0.87)
	Other family member	0.06	0.13	37 (0.13)	0.14	0.13	40 (0.13)
Father helping in home garden^**‡**^	Yes	0.67	0.54	158 (0.55)	0.50	0.46	146 (0.47)
Children assisting in home garden^**‡**^	Yes	0.22	0.21	62 (0.22)	0.21	0.31	89 (0.29)
Money received for snacks	Yes	0.35	0.34	112 (0.34)	0.27	0.21	71 (0.22)

*prop* Proportion

^±^A base package of R-software was used to perform the chi-square test (Pearson’s). However, no statistical significance was found (for a comprehensive table, see supplementary table 1)

^†^Literate: those who can read and write

^**‡**^the total number of samples is 289 and 307 in the control and treatment groups, respectively

^*^total number of PDs for variables (^**‡**^) is 18

For the treatment group, girls made up 55% of the schoolchildren in the sample. More than half (63%) were from Adivasi/Janajatis; approximately 8% were from Dalits. About 58% of parents could not read and write, and most were farmers (82%). Vegetables were produced by 97% of the households in home gardens, and mothers (87%) were main person responsible for taking care of the home gardens.

Table 2 shows the frequency of the seven food groups consumed by PDs and non-PDs. The schoolchildren consumed food from “grains, roots, and tubers.” The least consumed food group was eggs, consumed by only 7.2%. The frequency of consuming “legumes and nuts,” dairy products, eggs, and flesh foods were different between PDs and non-PDs (*p* < 0.05). Compared with non-PDs, PDs were more likely to consume “legumes and nuts” (odds ratio [OR] = 8.62; 95% confidence interval [CI]:1.14 – 64.95), dairy products (OR = 4.96; 95% CI:1.94 to 12.66), eggs (OR = 5.71; 95% CI:2.01 to 16.23), and flesh foods (OR = 3.02; 95% CI:1.27 to 7.21).

**Table 2:** Food consumption (proportion of schoolchildren) by positive (*n* = 23) and non-positive deviants (*n* = 309) in the control group^†^

Food groups	Positive deviants	Non-positive deviants	Odds ratio	95% CI
Grains, roots, and tubers	1.00	1.00	-	
Legumes and nuts	0.96	0.72	8.62^*^	1.14, 64.95
Dairy products	0.35	0.10	4.96^***^	1.94, 12.66
Eggs	0.26	0.06	5.71^***^	2.01, 16.23
Flesh foods	0.61	0.34	3.02^*^	1.27, 7.21
Vitamin-A rich fruits and vegetables	0.48	0.31	2.06	0.88, 4.85
Other fruits and vegetables	0.57	0.59	0.92	0.39, 2.16

*CI* confidence interval

^†^The *epiR* package [34] of R software was used to perform the chi-square test (Mantel–Haenszel) and odds ratio (Wald); significance

^*^ < 0.05

^**^ < 0.01

^***^ < 0.001. For a comprehensive table, see supplementary table 2

Table 3 shows the seven food groups consumed by the NDs and non-NDs. In the treatment group, all schoolchildren consumed “grains, roots, and tubers.” Similar to the control group, eggs were the least consumed by the schoolchildren. The frequency of consuming “legumes and nuts” and “other fruits and vegetables” were significantly different between NDs and non-NDs. Relative to non-NDs, NDs were less likely to consume “legumes and nuts” (OR = 0.49; 95% CI: 0.28—0.86) and dairy product (OR = 0.17; 95% CI: 0.04 to 0.74). In contrast, NDs were more likely to consume “other fruits and vegetables” (OR = 1.87; 95% CI:1.05 to 3.32).

**Table 3:** Food consumption (proportion of schoolchildren) by negative deviants (*n* = 73) and non-negative deviants (*n* = 244) in the treatment group^†^

Food groups	Negative deviants	Non-negative deviants	Odds ratio	95% CI
Grains, roots, and tubers	1.00	1.00	-	
Legumes and nuts	0.60	0.75	0.49^*^	0.28, 0.86
Dairy products	0.03	0.14	0.17^*^	0.04, 0.74
Eggs	0.04	0.05	0.76	0.21, 2.75
Flesh foods	0.45	0.43	1.07	0.63, 1.82
Vitamin-A rich fruits and vegetables	0.30	0.37	0.74	0.42, 1.30
Other fruits and vegetables	0.73	0.59	1.87^*^	1.05, 3.32

*CI* confidence interval

^†^ The *epiR* package [34] of R software was used to perform (Mantel–Haenszel) chi-square test and (Wald) odds ratio; significance

^*^ < 0.05

^**^ < 0.01. For a comprehensive table, see supplementary table 3

Table 4 shows factors associated with being PDs (control group) and NDs (treatment group) as identified using simple and multiple (Firth’s) logistic regression. In simple logistic regression, number of vegetables and fruits grown was negatively, and parents’ agricultural knowledge was positively associated with being PDs. After controlling for other covariates, schoolchildren’s eating frequency was positively associated with being PDs. Those who ate more frequently in a day (adjusted odds ratio, AOR = 2.25; 95% CI:1.07 to 5.68) and those whose parents had higher agricultural knowledge (AOR = 1.62; 95% CI:1.11 to 2.34) were more likely to be PDs.

**Table 4:** Factors associated with being positive deviants (control group) and negative deviants (treatment group) among schoolchildren

**Explanatory variables**	**Control group *n* = 332**				**Treatment group *n* = 317**			
	**OR**	**95% CI**	**AOR**	**95%CI**	**OR**	**95%CI**	**AOR**	**95% CI**
**Child characteristics**								
Schoolgirl (Ref. schoolboy)	1.24	0.53, 3.06	1.18	0.49, 2.95	0.86	0.51, 1.46	1.04	0.58, 1.87
Child < 10 years old (Ref. Child > 10 years old)	0.86	0.30, 2.14	1.02	0.35, 2.75	1.17	0.67, 2.03	1.03	0.55, 1.92
Knowledge of food and nutrition	0.91	0.60, 1.38	0.98	0.61, 1.56	1.01	0.77, 1.32	1.06	0.78, 1.43
Agricultural knowledge	1.20	0.80, 1.75	1.15	0.76, 1.70	1.21	0.92, 1.58	1.24	0.93, 1.66
Vegetable preference	0.94	0.62, 1.45	0.79	0.50, 1.26	0.87	0.66, 1.12	0.78	0.58, 1.04
Snack choice	0.88	0.58,1.35	0.88	0.56, 1.36	0.84	0.65, 1.09	0.87	0.66, 1.15
Frequency of eating	2.21	1.03, 5.77	2.25^*^	1.07,5.68	0.98	0.72, 1.37	1.02	0.73, 1.46
Consuming diverse vegetables	1.03	0.61, 1.72	0.99	0.56, 1.73	0.58^**^	0.41, 0.81	0.56^**^	0.38, 0.81
**Parent characteristics**								
Age (years)	1.03	0.97, 1.08	1.03	0.97, 1.09	0.99	0.96, 1.03	1.01	0.97, 1.05
Years of education	0.91	0.79, 1.02	1.09	0.92, 1.27	1.06	0.25, 1.06	1.03	0.94, 1.12
Main occupation farming (Ref. Not farmer)	1.02	0.39, 3.17	1.31	0.43, 4.47	0.84	0.44, 1.69	0.54	0.25, 1.19
Vegetable preference	0.76	0.48, 1.18	0.69	0.41, 1.12	0.88	0.67, 1.14	0.72^*^	0.53, 0.97
Food and nutrition Knowledge	0.81	0.54, 1.22	0.74	0.48, 1.17	1.26	0.96, 1.67	1.35	0.99, 1.86
Agricultural Knowledge	1.60^*^	1.07, 2.25	1.62^*^	1.11, 2.34	1.21	0.92, 1.58	1.11	0.83, 1.48
**Household characteristics**								
Family members < 6 (Ref. family member > 5)	0.74	0.31, 1.78	0.94	0.37, 2.48	0.81	0.48, 1.41	0.96	0.50, 1.85
Household having grandmother (Ref. No grandmother)	1.30	0.45, 3.27	1.08	0.36, 2.97	1.77 ^*^	1.00, 3.10	1.98^*^	1.03, 3.81
Food practices	0.91	0.61, 1.40	1.28	0.79, 2.17	1.12	0.87, 1.47	1.08	0.79, 1.50
Number of vegetables and fruits produced	0.80^*^	0.65, 0.98	0.84	0.64, 1.07	0.93	0.81, 1.06	0.89	0.76, 1.05
Number of days vegetables bought per week	1.18	0.98, 1.40	1.13	0.90, 1.42	0.84	0.70, 0.99	0.71^**^	0.56, 0.88
Dalits (= 1) (Ref. Brahaman/Chhetri)	2.83	0.53, 13.48	2.51	0.44, 13.59	0.49	0.11, 1.60	0.63	0.14, 2.19
Adivasi / Janajatis (= 1) (Ref. Brahaman/Chhetri)	2.72	0.97,9.69	2.35	0.72, 9.29	1.11	0.63, 2.02	1.45	0.76, 2.83

*OR* odds ratio, *CI* confidence interval, *AOR* adjusted odds ratio; The base package (*glm*) of Rsoftware was used to perform simple logistic regression, and *the Logistf* package [35] was used for firth logistic regression; significance

^*^ < 0.05

^**^ < 0.01

For the treatment group, the result of simple logistic regression shows that the consumption of diverse vegetables was negatively associated with being ND. However, the presence of grandmothers was positively associated with being ND. In the multiple (Firth’s) logistic regression analysis, after controlling for covariates, those schoolchildren who consumed diverse vegetables (AOR = 0.56; 95% CI: 0.38 to 0.81), had higher vegetable preference among parents (AOR = 0.72; 95% CI: 0.53 to 0.97), and had a higher number of days vegetables were bought per week (AOR = 0.71; 95% CI: 0.56 to 0.88), were less likely to be ND. Schoolchildren with grandmothers at home were more likely to be ND (AOR = 1.98; 95% CI: 1.03 to 3.81).

### Qualitative results

Among the parents interviewed, one parent in the PDs group and three in the NDs group were fathers, one person in the ND group was a grandmother, and all others were mothers. Further, four girls and five boys were interviewed from the PDs. Likewise, seven girls and two boys were interviewed among the NDs. All themes that emerged are compiled in additional file 4.

### Theme 1: Availability of diverse foods from home gardens and shops

Most interviewees (PDs and NDs parents) cultivated cereals and different kinds of vegetables. However, other food groups were either produced less or not produced at all. In addition, PDs can access food from markets, neighbors, or relatives. Some PD interviewees started a small shop, which increased the accessibility of diverse food groups:

“Now I migrated down (near road) and had a small shop… I have kept all vegetables for selling… that is (for selling and consumption) why I also cultivate coriander, radish, carrot, garlic, potato…” — (PD8; mother, 31 years old)

Willingness to buy or produce food also promotes food consumption. In addition to availability and accessibility, parents of PDs are willing to provide diverse foods for their children.

### Theme 2: Involving children in meal preparation

In rural areas in Nepal, the preparation of diverse food is time-consuming. ND interviewees expressed that the lack of adequate time was a barrier. Relative to parents of NDs, parents of PDs mentioned that their children contribute to the meal preparation, which may save parents’ time. Moreover, children could cook diverse foods:

“Children cooked yesterday… they cooked green leafy and green beans, vegetables, rice, and porridge.”* —* (PD4; mother, 36 years old)

Further, parents of PDs also mentioned that, as their children are involved in the meal preparation, they have more liberty to choose their preferred vegetables and food and are therefore more likely to eat it.

### Theme 3: Decision-maker regarding the food to be cooked

In most communities, parents decide what food to prepare. In the interview, all NDs interviewees mentioned that parents or grandparents decide what to prepare. In contrast, PD interviewees indicated that children also take part in food decisions.

“Sometimes, my mother [decides] what to cook… and, sometimes, we decide what to cook…” — (PDc7; girl, 14 years old, child)

### Theme 4: People’s knowledge of the benefits of diverse food

Most interviewees had some idea about the benefit of consuming diverse and healthy food, reflecting their declarative knowledge (knowledge of the content and things, for example, knowledge of green leafy vegetables helps to keep eyes healthy). However, an ND interviewee (grandmother) noted that she did not know about the benefits of consuming diverse foods:

“I do not know what [comprises] healthy foods… and I have no idea about unhealthy foods… I heard that we must consume legumes and green leafy vegetables, but I do not know what it does…” — (ND9: grandmother, 70 years old).

### Theme 5: Child preference for various foods

Most ND interviewees indicated that children disliked some food groups. It was also mentioned that children were picky and asked for money to buy junk food. In contrast, PD schoolchildren were not as picky. Moreover, parents of PDs (contrast to NDs) mentioned that they employed different techniques to make their children healthy, which may reflect that parents of PDs have some procedural knowledge (knowledge of how to do things, for example, how to cook food or how to select healthier snacks):

“He does not like beans; I advise him to eat beans… I must add potato with beans… he [does not] prefer beans… When I say it’s good to eat […]… he eats… he has some degree of preference only”* —* (PD2; mother, 33 years old)

Moreover, PD interviewees consumed locally-available stinging nettles available throughout the year.

### Theme 6: Parents’ attitudes and behavior toward their children’s food habit

Parents of PDs indicated that they tried not to buy junk food for children (although they prefer it sometimes) or families. Moreover, parents tried to motivate children to buy healthy foods by inculcating healthy behavior;

“We do not eat noodles… and we also never prepare noodles for them… I also do not eat noodles; […] I drink tea or milk… [not] noodles…” — (PD7; mother, 36 years old, mother)

In contrast, parents of NDs did not show resistance to their children’s preference for junk food.

### Theme 7: Food and feeding culture in the community

People from a higher ethnic group, such as Brahmin/Chettri, do not consume pork or buffalo meat. However, a PD interviewee said that this culture was prevalent among the older generation. Moreover, interviewees mentioned that as the new generation learned about healthy diets, they became more caring for children than the older generation;

“In the past, people did not care about children… they did not know what was good to feed their children… Now, even […] the village has changed; I think this [change] is because of the information they heard.” — (PD2; mother, 33 years old)

### Integration of quantitative and qualitative results

**Fig. 3:**
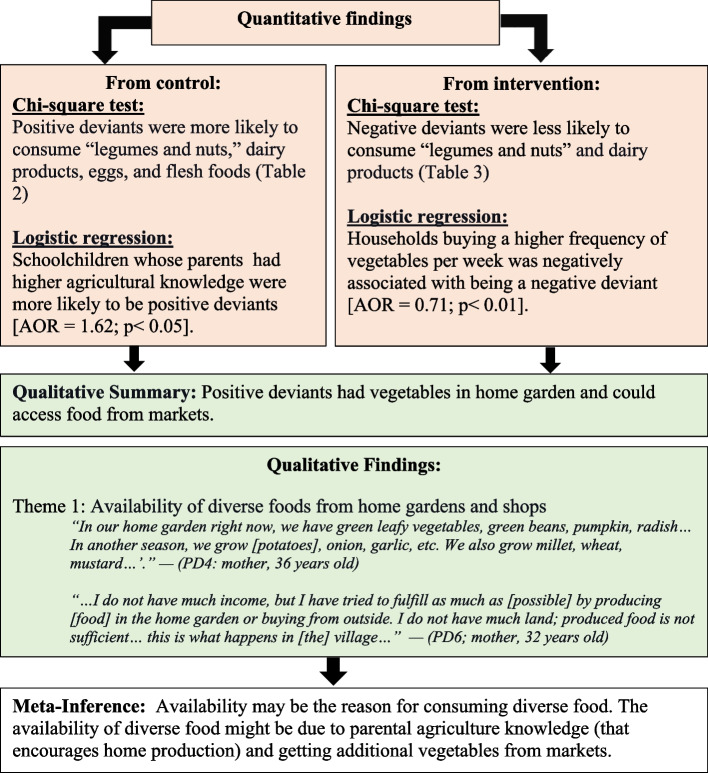
Availability of food groups improves dietary diversity

The integration of quantitative and qualitative data generated four main findings. First, schoolchildren who consumed purchased foods in addition to homegrown foods had a higher DDS (Fig. 3. Availability of food groups improves dietary diversity). The quantitative findings showed that better parental agricultural knowledge and more frequent buying of vegetables were associated with a higher DDS in schoolchildren. The qualitative findings also confirmed that the PD interviewees can access food from their home garden as well as from the market.

**Fig. 4:**
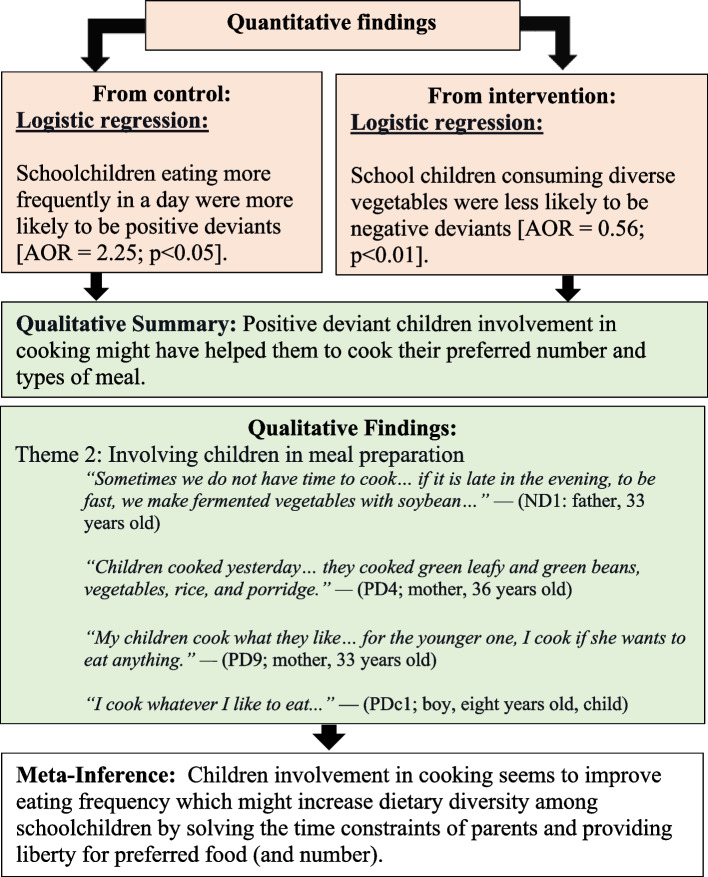
Child involvement in meal preparation improves dietary diversity

Second, the involvement of children in meal preparation was associated with a higher DDS among schoolchildren (Fig. 4. Child involvement in meal preparation improves dietary diversity). The quantitative data also indicated that eating more frequently was associated with being PDs, and consuming diverse vegetables was negatively associated with being NDs. The qualitative data showed that lack of time for cooking was a major reason for not preparing diverse foods. Moreover, PD schoolchildren were more involved in meal preparation, which helped them prepare their preferred food or vegetables.

**Fig. 5:**
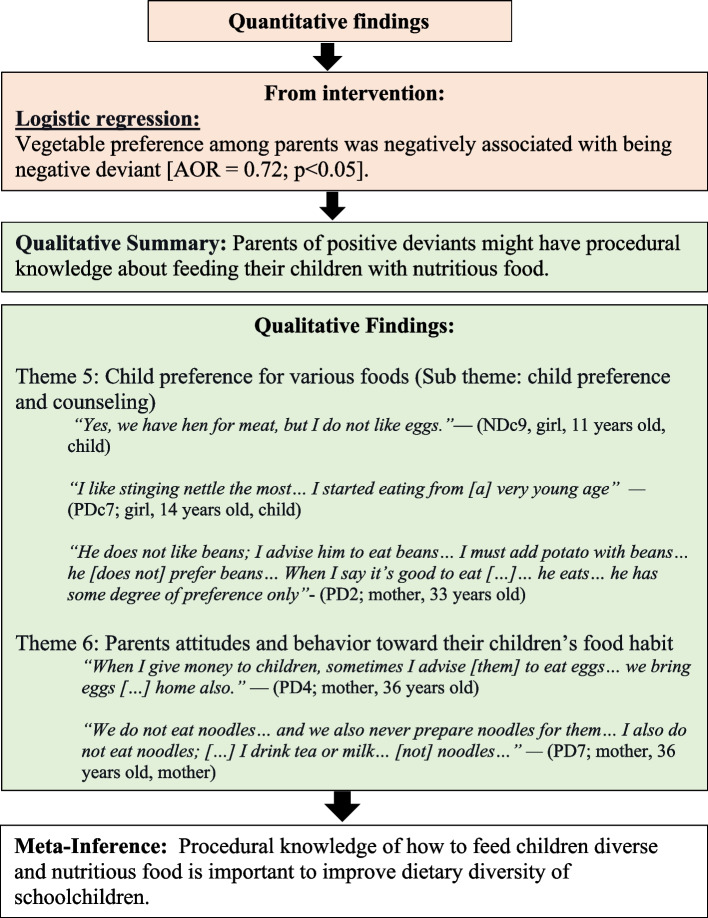
Parent’s procedural knowledge of food and nutrition, improves dietary diversity

Third, more procedural knowledge about food and nutrition among parents was associated with a higher DDS among schoolchildren (Fig. 5. Parent’s procedural knowledge of food and nutrition, improves dietary diversity). The quantitative results showed that vegetable preference among parents was negatively associated with ND. The qualitative findings showed that parents of PDs had procedural knowledge. Moreover, interviewees mentioned that they used procedural knowledge, such as different child-counseling methods on eating home-based foods, and even introduced vegetables (stinging nettle) that are nutritious and freely available throughout the year.

**Fig. 6:**
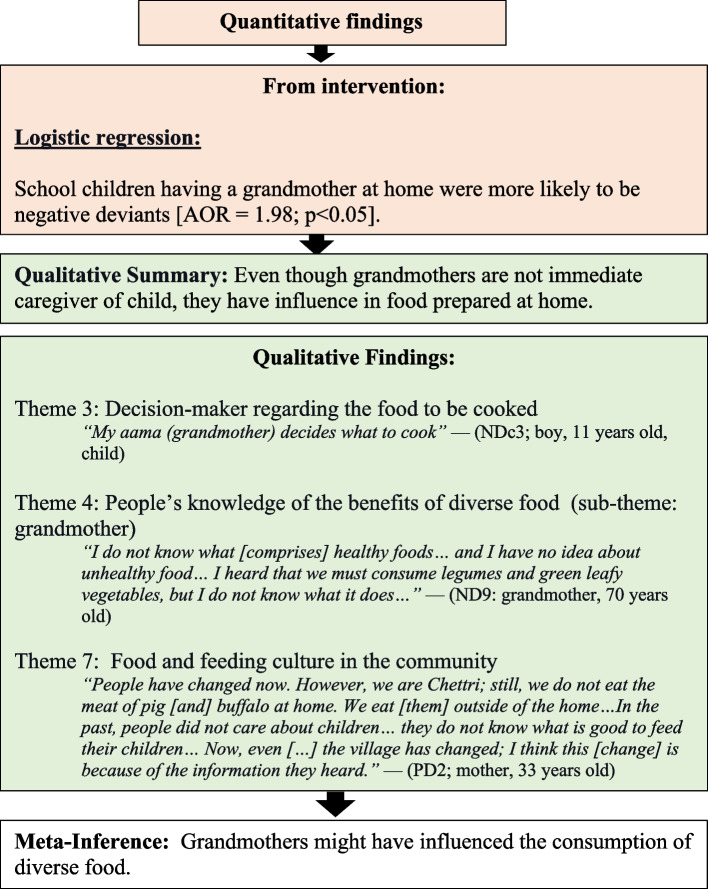
Influence of grandmother on schoolchildren’s dietary diversity

Fourth, the presence of a grandmother in the household is negatively associated with the consumption of diverse foods (Fig. 6. Influence of grandmother on schoolchildren’s dietary diversity). The quantitative results showed that schoolchildren with grandmothers at home were more likely to be NDs. The qualitative findings also showed that grandmothers might be comparatively poor in knowledge, decision-makers, and involved in the cooking. Furthermore, qualitative findings showed no better child feeding knowledge among the older generation than the present generation.

## Discussion

This study identified 23 PD and 73 ND schoolchildren, and four major factors that impact the consumption of diverse food among the schoolchildren. Greater availability of food of multiple food groups, children’s participation in meal preparation, parents’ procedural knowledge of food and nutrition, and the presence of grandmother impacted the consumption of diverse food among the schoolchildren.

Less than 30% of schoolchildren in this study had a minimum DDS in both the control and treatment group, which was less than the national data (above 40%) [36]. However, the national data show the lowest DDS in the mountain region [36], where our study site was situated. A majority of schoolchildren consumed starchy staple foods, which are similar throughout Nepal and countries such as Bangladesh, India, and Sri Lanka [21, 37, 38]. Moreover, NDs consumed more “other fruits and vegetables” than non-NDs, which might be due to the intervention though their overall DDS was still lower. From the food consumption patterns it shows that the schoolchildren consumed the basic diets consisting cereals and vegetables, which is similar to the consumption patterns among women in Tanzania [30].

The first integrated finding was that the increased availability of different food groups might be the reason for the improvement in DDS. This study showed that parental agricultural knowledge was associated with PD. Parents with good knowledge are likely to be involved in agriculture, thereby improving food security, increasing food diversity, and ultimately improving nutrition [29, 39]. A nationally representative study conducted in Nepal showed that agricultural diversity with different food groups was associated with children’s higher DDS among poorer households [40]. However, for a household with a small farm, self-produced food diversity cannot influence dietary diversity; accessibility to the market and purchasing or getting additional vegetables improve food diversity [30, 41]. Similar to the findings of Tanzania and Malawi [30, 41], this study found that schoolchildren whose households bought vegetables more frequently were more likely to have higher DDS. Moreover, the study has shown that parents of PDs were more willing to produce and buy vegetables.

The second integrated finding was the benefit of involving the child in meal preparation, which might improve DDS. A systematic review showed that children involved in meal preparation have more positive preferences, attitudes, and behavior toward healthy food [42]. Children involved in cooking have a higher preference for vegetables [43], leading to the consumption of diverse vegetables. In the Nepalese context, parents in a rural setting are usually busy with their day-to-day work. Therefore, in addition to improving preferences, attitudes, and behavior, children’s involvement in cooking could help prepare a higher frequency of meals. The increase in the number of meals can lead to a higher frequency of eating and improving schoolchildren’s dietary diversity, as found in the case of Ethiopia [44].

The procedural knowledge of parents was another important integrated finding that might increase schoolchildren’s DDS. The quantitative study showed that less vegetable preference among parents was associated with being NDs. The availability of different food groups might not be sufficient for the dietary consumption of diverse foods; accordingly, there should be a habit of consuming healthy foods. The literature has suggested that parents’ food habits majorly impact children’s food choices and eating behavior [45]. The indicator used (quantitative) to measure nutrition knowledge by the project were primarily comprised of declarative knowledge [14, 46]. The parents of PD interviewees mentioned that they used different techniques (considered as procedural knowledge), such as mixing food, not buying junk food for children, and teaching their children to consume nutritious food from an early age. The primary notion is that nutrition knowledge (declarative) is necessary but not sufficient to change nutritional behavior. More importantly, there should be additional procedural knowledge to change the behavior [46].

The final integrated finding was that grandmothers might influence the consumption of diverse foods. The quantitative data showed that schoolchildren with grandmothers at home were more likely to be NDs. The qualitative data also showed that even though grandmothers are not primary caregivers, they are involved in decision-making and food preparation. Moreover, they are more rigid with old cultures and customs. Children living with three-generational family members have a significant influence on their eating behavior [47]. Socio-cultural norms greatly impact grandparents’ feeding habits [48]. These norms were passed from grandparents to parents and from parents to children. A study conducted in Nepal on infant- and young-child-feeding found that households with grandmothers having correct nutrition knowledge were fed well relative to those with incorrect nutrition knowledge [49].

## Strength and limitations of the study

This study employed mixed methods and identified deviants and their behaviors, providing a better understanding of the dietary behaviors of the “school and home garden project” parents and schoolchildren. It can provide valuable information for future programs.

However, these results must be interpreted considering several limitations. Primarily, the study employed cross-sectional endline data, and causal inference cannot be interpreted between the outcome and determinants. The DDS was calculated based on self-reported 24-h recall consumption data; thus, the information may be subjected to day-to-day variability and social desirability bias. The project did not measure the food portion. Hence, to minimize the bias, we excluded food consumed in meager quantities, such as sesame seeds mixed in a pickle, milk tea, and ice cream (mostly made with powder in Nepal). Anthropometric and biomarker (for micronutrient) data of schoolchildren were not obtained, limiting the precise linkage of DDS and nutritional status of schoolchildren. Nonetheless, the study method to measure DDS has been widely used [50, 51].

Moreover, in Nepal, DDS varied among different age groups by different ecological regions and rurality [36]; this finding cannot be generalized to other areas. In the context of PD studies, generalizability could sometimes be a limitation; however, PD interventions are a problem-solving approach for a particular community [52].

## Conclusions

Schoolchildren’s involvement in meal preparation at home and raising awareness among family members is necessary to improve schoolchildren’s dietary diversity score (DDS). These findings could provide vital information for designing and implementing future research and programs, thus improving the DDS of schoolchildren in resource-limited settings. The findings were obtained by focusing on positive and negative deviants. Similar efforts may be applied to other health studies.

## Supplementary Information

**Additional file 1.** 

**Additional file 2.** 

**Additional file 3.** 

**Additional file 4.** 

**Additional file 5:**

## Data Availability

The datasets supporting the conclusions of this article are available in the https://dataverse.harvard.edu/privateurl.xhtml?token=ae6a2eda-d078-46ed-8018-9c9a114d7216

## Abbreviations

AOR: Adjusted Odds Ratio
CI: Class Interval
DDS: Dietary Diversity Score
ND: Negative Deviant
OR: Odds Ratio
PD: Positive Deviant
