# Supplementary material for: Identifying positive and negative deviants and factors associated with healthy dietary practices among young schoolchildren in Nepal: a mixed methods study
Source: BMC Nutr. 2023 Mar 8;9:42. doi: 10.1186/s40795-023-00700-5 (PMC9993389; doi:10.1186/s40795-023-00700-5)
Supplement: Supplementary file 1 — Additional file 1. [file 40795_2023_700_MOESM1_ESM.docx]

**Additional file 1**

**Outline of implemented school and home garden project**

The “school and home garden” project was a cluster-randomized controlled trial (RCT), conducted in 30 schools in Sindhupalchok district of Nepal from January 2018 to December 2019^(1)^. The district has an area of about 2542 km^2^ and spread from mid-hills to mountain region and closer to Kathmandu valley.

The project hypothesized that adding a household garden to previously conducted school garden project^(1,2)^, may improve healthy dietary habits. Program theory of the intervention is attached below in Figure 1.


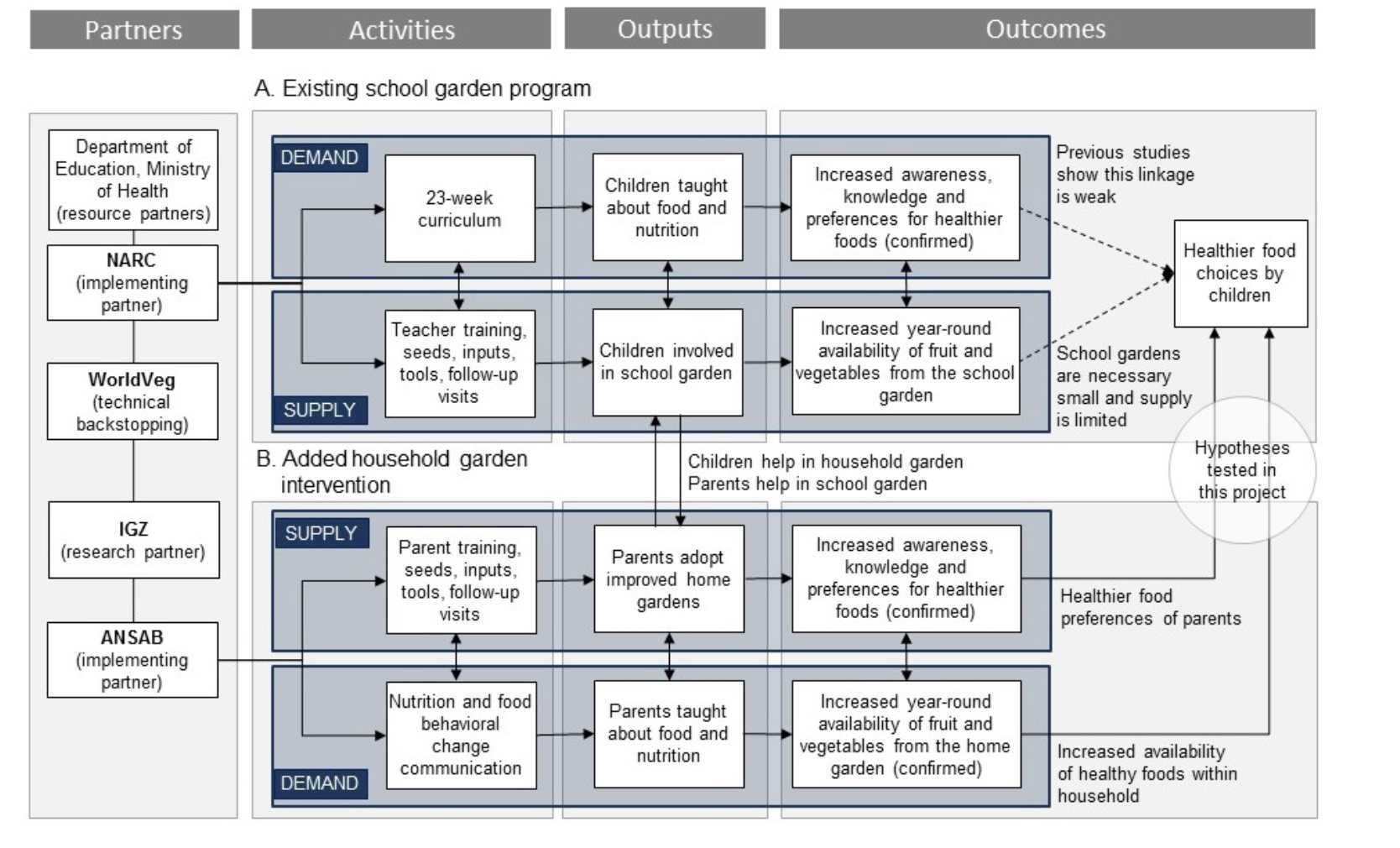


**Supplementary figure 1** Program theory of the intervention tested in the school and home garden project.

*Source: proposal of the “school and home garden project”*

.

The study included two arms (15 schools in control, and 15 schools in intervention).

The sample size was calculated as 450 schoolchildren and their caregiver in each group (control and intervention). The detail of sample calculation and selection process was mentioned in the previous study^(1)^.

**The questionnaire**

The baseline and end-line survey of the project employed two sets of the questionnaire for schoolchildren and their parents, respectively. For schoolchildren’s survey, assisted self-administered questionnaires were used which included photographs and minimal text to record children’s food and nutrition knowledge^(1–3)^, agricultural knowledge^(1)^, vegetable preferences^(1,2,4)^, snack choices^(1,5)^, and general information (name, age, gender, student number). A logbook was maintained for 24-hours recall for food intake. Dietary diversity score, vegetable score (number of meals containing vegetables), and vegetable diversity (types of vegetables consumed in a day) were calculated from the logbook. For the parents’ survey, a face-to-face interview was conducted using a structured questionnaire. The questionnaire related to knowledge and preference was similar to children’s questionnaire. In addition, it included information such as food practices, socio-demographic, household assets, home-garden, and vegetable production.

**References**

1. Schreinemachers P, Baliki G, Shrestha RM, et al. (2020) Nudging children toward healthier food choices: An experiment combining school and home gardens. *Glob. Food Sec* **26**, 100454.

2. Schreinemachers P, Bhattarai DR, Subedi GD, et al. (2017) Impact of school gardens in Nepal: a cluster randomised controlled trial. *J Dev Eff* **9**, 329–343.

3. Oldewage-Theron WH & Egal AA (2010) Nutrition knowledge and nutritional status of primary school children in QwaQwa. *South African J Clin Nutr* **23**, 149–154.

4. Heim S, Stang J & Ireland M (2009) A Garden Pilot Project Enhances Fruit and Vegetable Consumption among Children. *J Am Diet Assoc* **109**, 1220–1226.

5. Lineberger SE & Zajicek JM (2000) School gardens: Can a hands-on teaching tool affect students’ attitudes and behaviors regarding fruit and vegetables? *Horttechnology* **10**, 593–597.
