# Supplementary material for: Identifying positive and negative deviants and factors associated with healthy dietary practices among young schoolchildren in Nepal: a mixed methods study
Source: BMC Nutr. 2023 Mar 8;9:42. doi: 10.1186/s40795-023-00700-5 (PMC9993389; doi:10.1186/s40795-023-00700-5)
Supplement: Supplementary file 2 — Additional file 2. [file 40795_2023_700_MOESM2_ESM.docx]

**Additional file 2**

**In-depth Interview procedure**

Initially, face to face in-depth interview was planned, however, due to the Covid-19 (there was lock down) plan was changed to phone in-depth interview. The PDs and NDs pairs were selected based on the availability of a phone number. The phone numbers of the interviewees were extracted from the baseline and end-line data of the “school and home garden” project.

Four research assistants (RAs)—three men and one woman conducted the interviews. The female RA had completed her undergraduate degree in public health and was working as a freelancer. All three male RAs were public health master’s students. All the RAs were trained and experienced in qualitative research.

Moreover, they were trained in a one-hour session to introduce the research, objective, nature of participants, procedure, and ethical consideration. The guidelines were pretested before the actual data collection and edited based on the pretest feedback.

Rapport building was done with the interviewees before the data collection. For rapport building, RAs contacted interviewees before the actual data collection. RAs asked the preferred time for an interview, and interviews were taken on the mentioned date and time. The phone interview was conducted one-on-one between RAs and interviewees. The interviewees were informed about the research and its objectives and ensured the confidentiality of the interview. Before starting the interview, the RAs recorded verbal informed consent from the interviewees. On average, each interview lasted about 30 to 45 minutes. The research assistants recorded the interviews on phones and laptops and took field notes for important information.

Interviewees stayed at home at the time of interventions, except for one schoolchild—she was at a maternal uncle’s home. We got consent and a number from the parents. While doing the interview, we asked if there were anyone around her and if she was comfortable talking. We interviewed her, and she assured us that she was comfortable talking to us independently. We interviewed separately. Data saturation was reached after conducting all nine pairs of interviews.
