# Supplementary material for: Identifying positive and negative deviants and factors associated with healthy dietary practices among young schoolchildren in Nepal: a mixed methods study
Source: BMC Nutr. 2023 Mar 8;9:42. doi: 10.1186/s40795-023-00700-5 (PMC9993389; doi:10.1186/s40795-023-00700-5)
Supplement: Supplementary file 3 — Additional file 3. [file 40795_2023_700_MOESM3_ESM.docx]

**Additional file 3**

**In-depth interview guideline for positive deviants and their parents (English)**

**Participants:** Parents of positive deviants

Introduction: Hello Namaste, I want to thank you for allocating the time to be interviewed by me through phone today. My name is……………………. and I would like to talk to you about your experiences about the diversified food and healthy eating which is related to my research topic.

The interview should take less than an hour. I will record the session because I don’t want to miss any of your comments. In addition, I will be writing some important notes during the session. Please be sure to provide us as much as information you can.

All responses will be kept confidential. This means that your responses will only be shared with research team members. We would like to ensure that any information we include in our report will not disclose your identity. Remember, you don’t have to talk about anything you don’t want to, and you may end the interview at any time.

Do you have any questions about what I have just explained?

Are you willing to participate in this interview?

Yes ……………………… No…………………

| **S. N** | **Major questions** | **Probes** |
| --- | --- | --- |
|  | What did you prepare for lunch today? | - Who cooked it? - Who usually cook in your household? |
| **Availability** | | |
|  | Do you grow all the food that you cook? | - Why and why not? - What food items do you grow (list)? (explore if they grow diverse food or not; try to explore reason) |
|  | Do you buy any food item from markets | - Why and why not? - Can you list what food items do you buy? (explore what food items, probing questionnaire can according to the answer; try to explore reason) |
|  | Most of the other children were unable to get more than 4 types of foods, how have you been able to manage to give more than 4 types in a day. | - What is the technique you do to fulfil the food demand of your family? - Do you buy or exchange any food items? |
| **Behaviour** | | |
|  | How many times do you cook food? | - Do you cook different each time? - Do you cook mixed food? - Why and why not |
|  | How many times does your family eat food? | - (explore if they eat breakfast, lunch, snacks and dinner) - Why and why not |
|  | Usually at what time does your household have a meal? | - Ask the time - Do you child eat lunch before going to school |
|  | Does your family eat together? | - why and why not |
|  | Do you provide some food rewards | - (can you list the food items) |
|  | Do you prepare snacks for your children?  (or provide money for snacks) | - Do you cook or buy snacks? - What do you cook for snacks? (list) - If you buy, what do you buy for snacks? (list) |
|  | Some of the children do not want to eat some kind of vegetables or fruits, how do you manage in this situation. | - Any example/technique |
| **Society/community/influence** | | |
|  | Let's talk about information about healthy eating? | - Do you get any information about the benefits of eating diverse food? - From whom/what (medium) - Do you get any information about the negative health impact of eating a non-healthy meal (add non-diverse food later) |
| **Culture** | | |
|  | Do you have any culture that helps your household to have diverse food? | - What is culture/food items? - Is that culture common to the whole community or for specific household/small community - how frequently |
| **Knowledge** | | |
|  | In your opinion, what is a “healthy meal”? | - Can you describe a dish that you consider healthy? - What makes a meal healthy? - Do you regularly eat such a meal?   If not, why? |
|  | Can you also give an example of an "unhealthy meal"? | - What makes a meal unhealthy? - How often do you eat such a meal? - if you know that certain foods are unhealthy, why do you eat it? |
|  | In your opinion, what are the benefits of diversified healthy food? | - Can you list the health benefit when we eat diversified food?   Can you list what happens when we do not eat diversified food? |
|  | Is there anything you would like to tell me about which I haven’t thought to ask you? | |

**Participants:** Positive deviant children

| **S. N** | **Major** | **Probes** |
| --- | --- | --- |
|  | What did you eat today before coming here/lunch? | - Who cooked it? - Who usually cook in your household? - Do you tell your mom what food you like to eat? - Does she listen to you? - Who then decides about what to eat? |
| **Availability** | | |
|  | Do you like all vegetables and fruits are grown in your home garden? | - Can you list - Why and why not? |
| **Behaviour** | | |
|  | Do you have any vegetables or fruits that you don't like, but you eat? | - Reason/example |
|  | How many times does your family eat food? (in school days) | - (explore if they eat breakfast, lunch, snacks and dinner) - Why and why not |
|  | Do you feel that you have a choice in what you eat? | - Can you give an example |
|  | Does your family eat together? | - why and why not |
|  | Do you get money for snacks? | - What do you buy? (list) |
|  | What do you usually bring for your snack? | - Can you mention some of the names of snacks? - If you could choose any of these snacks, which one would you choose, and why? - Would most other children in your class choose the same? Why or why not? |
| **Society/community/influence** | | |
|  | Let's talk about information about healthy eating? | - Do you get any information about the benefits of eating diverse food? - From whom/what (medium) - Do you get any information about the negative health impact of eating a non-healthy meal (add non-diverse food later) |
| **Knowledge** | | |
|  | In your opinion, what is a “healthy meal”? | - Can you describe a dish that you consider healthy? - What makes a meal healthy? - Do you regularly eat such a meal? - If not, why? |
|  | Can you also give an example of an "unhealthy meal"? | - What makes a meal unhealthy? - How often do you eat such a meal? |
|  | In your opinion, what are the benefits of eating diversified foods? | - Can you list the health benefit of eating diversified food? - Can you list what happens when we do not eat diversified food? |
|  | - Is there anything you would like to tell me about which I haven’t thought to ask you? | |

**Participants:** Parents of negative deviants

Introduction: Hello Namaste, I want to thank you for allocating the time to be interviewed by me through phone today. My name is……………………. and I would like to talk to you about your experiences about the diversified food and healthy eating which is related to my research topic.

The interview should take less than an hour. I will record the session because I don’t want to miss any of your comments. In addition, I will be writing some important notes during the session. Please be sure to provide us as much as information you can.

All responses will be kept confidential. This means that your responses will only be shared with research team members. We would like to ensure that any information we include in our report will not disclose your identity. Remember, you don’t have to talk about anything you don’t want to, and you may end the interview at any time.

Do you have any questions about what I have just explained?

Are you willing to participate in this interview?

Yes ……………………… No…………………

| **S.N** | **Major questions** | **Probes** |
| --- | --- | --- |
|  | What did you prepare for lunch today? | - Who cooked it? - Who usually cook in your household? |
| **Availability** | | |
|  | Do you grow all the food that you cook? | - Why and why not? - What food items do you grow (list)? |
|  | Do you buy any food item from markets | - Why and why not? - Can you list what food items do you buy? |
|  | Diverse food is important for a healthy life. But sometimes we are unable to provide more than 4 types of foods to our children, what are the reason do you think. | - Can you list the reasons? Why |
| **Behavior** | | |
|  | How many times does your family eat food? | - (explore if they eat breakfast, lunch, snacks and dinner) - Why and why not |
|  | Usually at what time does your household have a meal? | - Ask the time - Do you child eat lunch before going to school |
|  | How many times do you cook food? | - Do you cook different each time? - Why and why not |
|  | Does your family eat together? | - why and why not |
|  | Do you provide any food rewards to your children | - If yes, can you list |
|  | Do you prepare snacks for your children? (prepare, buy or money) | - Do you cook or buy snacks? - What do you cook for snacks? (list) - What do you buy for snacks? (list) |
| **Society/community/influence** | | |
|  | Let's talk about information about healthy eating? | - Do you get any information about the benefits of eating diverse food? - From whom/what (medium) - Do you get any information about the negative health impact of eating a non-healthy meal (add non-diverse food later) |
| **Culture** | | |
|  | Do you have any culture/restriction that prohibits your household from having diverse food? | - What is the culture/ what are the food items? - Is that culture common to the whole community or for specific household/small community |
| **Knowledge** | | |
|  | In your opinion, what is a “healthy meal”? | - Can you describe a dish that you consider healthy? - What makes a meal healthy? - Do you regularly eat such a meal? - If not, why? |
|  | Can you also give an example of an "unhealthy meal"? | - What makes a meal unhealthy? - How often do you eat such a meal? - if you know that certain foods are unhealthy, why do you eat it? |
|  | In your opinion, what are the benefits of diversified food? | - Can you list what the health benefit of eating diversified food are - Can you list what happens when we do not eat diversified food? |
|  | Is there anything you would like to tell me about which I have not thought to ask you? | |

**Participants:** Negative deviants

| **S.N** | **Major Questions** | **Probes** |
| --- | --- | --- |
|  | What did you eat today before coming here/lunch? | - Who cooked it? - Who usually cook in your household? - Do you tell your mom what food you like to eat? - Does she listen to you? - Who then decides about what to eat? |
| **Availability** | | |
|  | Do you like vegetables and fruits grown in your home garden? | - Can you list (like/dislike) - Why and why not? |
|  | Do you buy any food item from markets | - Why and why not? - Can you list what food items do you buy? |
|  | Diverse food is important for a healthy life. But sometimes we are unable to consume more than 4 types of foods, what are the reason do you think. | Can you list the reasons? Why |
| **Behavior** | | |
|  | How many times do you eat food? | - (explore if they eat breakfast, lunch, snacks and dinner) - Why and why not |
|  | Do you feel that you have a choice in what you eat? | - Can you give an example? - Why |
|  | Does your family eat together? | - why and why not |
|  | What do you usually bring for your snack? | - What are the famous snacks that children bring to school? - Can you name some snacks that you bring to school? - If you could choose any of these snacks, which one would you choose, and why? - Would most other children in your class choose the same? Why or why not? |
|  | Do your parents provide you with food rewards | Can you list them |
| **Society/community/influence** | | |
|  | Let's talk about information about healthy eating? | - Do you get any information about the benefits of eating diverse food? - From whom/what (medium) - Do you get any information about the negative health impact of eating a non-healthy meal (add non-diverse food later) |
| **Knowledge** | | |
|  | In your opinion, what is a “healthy meal”? | - Can you describe a dish that you consider healthy? - What makes a meal healthy? - Do you regularly eat such a meal? - If not, why? |
|  | Can you also give an example of an "unhealthy meal"? | - What makes a meal unhealthy? - How often do you eat such a meal? - If you know that certain foods are unhealthy, why do you eat it? |
|  | In your opinion, what are the benefits of diversified food? | - Can you list what health benefit when we eat diversified food? - Can you list what happens when we do not eat diversified food? |
|  | Is there anything you would like to tell me about which I haven’t thought to ask you? | |
