# Supplementary material for: Identifying positive and negative deviants and factors associated with healthy dietary practices among young schoolchildren in Nepal: a mixed methods study
Source: BMC Nutr. 2023 Mar 8;9:42. doi: 10.1186/s40795-023-00700-5 (PMC9993389; doi:10.1186/s40795-023-00700-5)
Supplement: Supplementary file 4 — Additional file 4. [file 40795_2023_700_MOESM4_ESM.docx]

**Additional file 4**

**Compiled themes generated from PDs group interview**

| **Themes** | **Codes** | **Quotes** |
| --- | --- | --- |
| Participatory cooking/children involvement | Family involvement | "In my house, anyone who have time can cook food. We are people from hills not from kathmandu, one person is allocated for cooking and another for work…....." PD1 |
|  | Male involvement | "I cooked today…..we cook turnwise, me and my husband…...." – PD7 |
|  |  | "usually I prepare food…my husband also prepare some time …..." – PD8 |
|  | Mother | "I don't work and my husband work…...until now it has helped us to fulfill food demand…I cooked rice, vegetables, pulses, pickle and eggs" PD2 |
|  |  | "…………. his (son’s) mother cooked food" – PD5 |
|  | Children involvement | "after I wake up I drink hot water, children cook rice and serve me. Then if there is remaining rice children eat in day….then in evening we make dhido ." PD1 |
|  |  | "My younger son is good in making chapati and vegetables…..when I make flour and ask him to make, he makes very well…" PD1 |
|  |  | "Children cooked yesterday….....they cooked green leafy and bodi vegetables, rice and *dhido*" – PD4 |
|  |  | "we ate green leafy vegetables, green beans , soyabean…......, my sister cooked it"-PDC4 |
|  |  | "yes I ate food, I cooked myself…...usually I cook myself"PD 6 |
|  |  | "my younger brother made pickel and other were prepared by my mother.." PD1 |
|  |  | " I cooked myself…...usually I cook myself"- PDc6 |
|  |  | "my younger brother made pickel and other were prepared by my mother.." PDc8 |
|  |  | "I cooked food…children went to village, so made it…..My kids also prepare foods food….I am teaching my kids….." PD9  "My children cook their-self what they like….for younger one I cook if she want to eat anything "- PD9  "Usually we both cook….today I came late from village so, she has cooked when I reached at home"- PDc9 |
|  | Benefit of children cooking | "I cook whatever I like to eat…..."- PDC1 |
| Availability/Accessibility for the different food groups | Food produced at home | "we have pumpkin, chayote squash, green leafy vegetables , green beans in our home garden…........" – PD1 |
|  |  | "we cultivate green leafy vegetables, garlic, coriander, raddish, garden cress, etc….....in season we also grow farsi ko munta, bodi, then karkalo…..”PD2 |
|  |  | "we live in village type of community…...even we buy, we can buy locally produced vegetables. We don't produce many vegetable, as we are busy….....we have cultivated onion and green leafy vegetables only, they are still growing"- PD3 |
|  |  | "In our home garden right now we have green lefy vegetables, bodi, pumpkin, raddish….... In other season we grow potato, onion, gralic.... We also grow kodo, wheat, mustard…." – PD4 |
|  |  | "we produce 95% of food what we eat, 5% we need to search outside.." - PD5  "we have green bean, bottle gourd, beans, brinjal, pumpkin, luffa gourd….I have vegetable farming also…......." – PD5 |
|  |  | "we cultivate kodo, maize….I sell goat and buy rice…....I have recently cultivated green leafy vegetables" - PD6  "right now we have cultivated kodo, green vegtables, green beans…......." – PD6  "yes we have green leafy vegetables, green beans, garlic, bean….."- PDc6 |
|  |  | "we have little bit land, we don't have much production. But we produce vegetabels and beans…...we cultivate rice but not wheat, and, we have maize and millet…...we don't have much fruits in our garden….. " - PD7  "we have cultiavted maize, and with in maize field we cultivated co-co yam…." – PD7  "we cultivate vegetable all around the house and in garden…..we have goat and some local chickens" – PD7  "we have cultivated chayote squash, leafy vegetables, bottle gourd…..we have banana and pomelo.." – PD7 |
|  |  | "we produce little bit vegetables… we don't have in winter season but in other season we have…...we cultivate cauliflower, green leafy vegetables, garlic, potato, raddish…....In village I didn't know about the cultivation….Now I came down and have small shop.....I have kept all vegetables for selling.....thats why I also cultivate corrinder, raddish, carrot, garlic, potato....." - PD8  "we produce rice and maize. We plant beans with maize plant…....but we don't have much land, so we do not cultivate millet" – PD8  "yes, we have home garden nearby…. right now we have tomato, cabbage, cauliflower and pumpkin…...yes I like those vegetables…....we have fruits in our shop…...and we have milk in my maternal uncle house…I like milk and yogurt " – PDc8 |
|  |  | "we have cultivated green beans…......we have mize and millet…..paddy is cultivated in village"- PD9  "yes we have lemon and orange in our …"- PD9 |
|  | Buying from the market | "we buy rice, pulses, oil, salt….., eggs, meat…........if our cow starts giving milk, we do not need to buy"-PD1 |
|  |  | "we buy egg, milk and meat…we also buy sponge gourd, bottle gourd etc…..........we have just cultivated cauliflower, so we need to buy"; PD2 |
|  |  | "we don’t produce much….we only cultivate kodo and maize…....my husband does job, and help run our shop…..we usually buy everything" - PD3  "Yes, my children want meat and we cook three time a week….and we also buy milk, yogurt…..we also eat green leafy vegetables" PD3 |
|  |  | "we usually don't buy vegetables….but we need to buy pulses, when we want to eat peas and beans we need to buy…they don't grow here….....we also buy fish, meat and eggs" – PD4 |
|  |  | "we need to buy potato in rainy season, in winter I cultivate potato…...I have buy in month of Ashar, shrawan, Bhadrea and Ashoj" - PD5  "we don't buy meat regularly…......if buy if we find in village…..we only buy daily consuming spices, oil…if children like some time they buy noodles, biscuits and bitten rice" – PD5  "we can't have diverse food in our village….we eat today one type tomorrow another type…..some time children wants to fried rice, we add soyabean, garlic…...we don't have every thing we need to eat in balance…."PD5  "…...but it is not possible in our village....we cannot buy what we need instantly….we don't have money too…however we eat some time balance diet" – PD5 |
|  |  | "we buy rice, salt, oil, spices, and, fish and meat… and we also buy peas and beans " – PD6  "I have not able to provide as much as children requirement…...i don't have much income but I have tried to fulfill as much as by producing in home garden or buying from outside. I don't have much land, produced food is not sufficient…this is what happens in village......." – PD6 |
|  |  | "we buy potato, salt, oil, spices and legumes from the market…...and we buy rice too..." – PD7 |
|  |  | "we have small shop…so we buy most of things…..For eating, we buy oil, spices, lentils…...we have vegetables, all other we need to buy, we don't have other option" – PD8 |
|  |  | "we buy black lentils…other lentils we have…..” PD9  "we don't need to buy until kartik and mangsir….....later we need to buy " – PD9 |
| Sharing of food | Sharing with neighbor | "some time we share foods with in our neighbour….....so some time we can get food, which we have not cultivated" PD2 |
|  | Sharing with neighbor | "when we have vegetables in our garden at the time of our neighbour's vist we give them too"- PD4 |
|  | Currently not sharing | "In past we used to share but now days we don't…......it is enough from our home production…....we sell surplus vegetable" – PD5 |
|  | Sharing with neighbor | "yes, we share food produced in our home garden with nearby neigbhour…..I give what we have, and we also exchange foods"- PD6  "we share what we have produced and they also give us…" – PD6 |
|  | No sharing at household near market | "we bring vegetables from village, we have small shops…….sharing could be find in faraway villages…...we sell and buy here" - PD3 |
|  | Not sharing | "we do not share food….we produce or sell" – PD7 |
|  | Sharing among relatives | "yes we share foods produce between sisters in law…..we give what they didn't have produce and they give us what we don't have…..." PD9 |
| Consuming behavior | Current consumption | " we ate rice and vegetables....................we ate chayote squash, green leafy vegetables, green beans …...........mixed" PD1  "I ate rice and vegetables…..i ate mix vegtables, chyote, beans, vegetables"- PDC 1 |
|  |  | "I cooked rice, vegetables, pulses, pickle and eggs"- PD2 |
|  |  | "I ate pulse, rice, vegetable, pickel,fry bitter gourd…......I ate sqush and potato mix vegetable"- PD3 |
|  |  | "we ate green leafy vegetables, green beans , soyabean…......, my sister cooked it" PDC4 |
|  |  | "today in luch we ate pulse, vegetables, rice…........" – PD5  "I ate pulse, rice and vegetable…....mom made it " – PDc5 |
|  |  | "I ate pulse and rice….... Green leafy vegetables were not grown yet" – PD6 |
|  |  | "today I cooked, potato and green leafy vegetables, and legumes….rice as usual"- PD7 |
|  |  | "we ate lentils, pickle, egg and rice" – PD8  "today we consume lentils, eggs and pickel…" – PD8 |
|  | Usual consumption | "we usually eat Kodo, maize, rice…..some time we eat chapati, but we eat different vegetables in lunch and dinner" PD1  *“….....*we often eat *taro leaf*" PD1 |
|  |  |  |
|  |  | "we usually cook pulses and vegetables…....and along with this my child don't want to eat rice without meat or egg" PD2 |
|  |  | "In our food, we mix green bean to green leafy vegetables and we cook pulses some time …..we sometime cook rice, didho, and chapati" – PD4 |
|  | Mix vegetable | "we usually prepare mix food…..we mix potato with beans…..we at least two types of vegetables " – PD5 |
|  |  | "whenever we cook vegetable, we cook mix vegetables.." |
|  |  | "we don't same food, we don't like eating. Some time we prepare green leafy vegetables, pulses, chayote squash, some time legumes, sometime cauliflower. We prepare what children like… they like legumes, potato, green leafy vegetables….... They like little bit meat, and they like milk and yogurt…...." PD7  "we cook meat little bit, vegetables with potato,and legumes …...and green leafy vegetables" – PD 7 |
|  |  | "we eat rice for both lunch and dinner…..we mix vegetables or prepare separately….both of them don't want to eat same food evey time…Now its season of vegetables, we have green leafy vegetrables. And children want meat or at evening we prepare legumes, lentils, eggs, potato....some time we prepare pumpkin" – PD8 |
| Parents behavior | Parents behaviour regarding junk food | "I have never bought noodles for children…..however, sometime we need to make children happy, so some time when I give them money they buy noodles"- PD1 |
|  |  | "when I give money to children, some time I advise to eat eggs…..we bring eggs to home also" – PD4 |
|  |  | "I have not able to provide as much as children requirement…...i don't have much income but I have tried to fulfill as much as by producing in home garden or buying from outside. I don't have much land, produced food is not sufficient…this is what happens in village”-PD6 |
|  |  | "we usually consume normal food… I heard there are many germs in meat….so we eat less meat and we consume legumes, green vegetables and furits…...we do not eat noodles….my children don't like noodles, and we also never prepare noodles for them.It is not good for health, it make thin to our body....my children prefer rice, pulses and vegetables than noodles.......I also do not eat noodles, despite I drink tea or milk....no noodles...." - PD7  "In our nepali food, healthy food are dhido , gundruk, green vegetables, stinging Nettle, Taro leaf…even stinging nettle is better… These all things are prepared by self….I think food prepared by self are better than those found in market"- PD7 |
| Eating together | Reason for eating together | "…..if we eat together with family, even food is not good, it tastes good. There is happiness when we eat togther…....we can see our children eating behaviour"- PD1 |
|  |  | "…..if we eat together with family, even food is not good, it tastes good. There is happiness when we eat togther…....we can see how much our child eat" – PD1 |
|  |  | "we are only three member….......so when we eat together it feels good and food also feels tasty" PD2 |
|  |  | "we do not eat lunch together, my child eat early and goes to school, then my husband, after that I eat last….......for dinner we eat together" – PD3 |
|  |  | "we eat food together…...it is easy to eat together"- PD4 |
|  |  | "we eat together, I don't know the reason but as we all are home, we eat together" – PD5 |
|  |  | " we some time eat lunch together and some time we don't….in morining if I am out for work then we can't eat together….but we eat dinner together" - PD6  "In morning we eat separately but in evening we eat together….... In morning my mother goes to work that’s why we eat separately"- PD6 |
|  |  | "we eat lunch separately with time interval of half to one hour…" – PD7  "we eat at around 9:00 pm, but children eat at around 7:00 pm and go to bed, they sleep early…." – PD7 |
|  |  | "we usually eat together…. if some time, if there is hurry, then at that time we give food to him and send to school…otherwise we eat together" – PD8 |
|  |  | "some time if we are hungry, we eat together. Otherwise, we first serve to children and then later we eat…. usually we eat together" – PD 9 |
| Parents preference | Prefer to consume diverse food | "we eat rice for both lunch and dinner…..we mix vegetables or prepare separately….both of them don't want to eat same food evey time…Now its season of vegetables, we have green leafy vegetrables. And children want meat or at evening we prepare legumes, lentils, eggs, potato....some time we prepare pumpkin" PD8 |
|  | Persistent cooking of healthy food | "I didn't used to eat stinging nettle….now a days I have changed bit of my habbit"- PDC1 |
|  | Diverse preference | "we usually have lentils at morining and vegetables at morining "- PDc9 |
|  |  | "we don't eat same food, we don't like eating. Some time we prepare green leafy vegetables, pulses, chayote squash, some time legumes, sometime cauliflower. We prepare what children like… they like legumes, potato, green leafy vegetables….... They like little bit meat, and they like milk and yogurt…...." PD7 |
| Children preference | Preference of specific food | "my children eat every thing…....but they less prefer *stinging nettle….....*we often eat *taro leaf*" PD1 |
|  |  | "we usually cook pulses and vegetables…....and along with this my child don't want to eat rice without meat or egg"- PD2 |
|  |  | " My children does not prefer same vegetables in morning and evening….......if he ate green vegetables in morning then in eveining we cook different vegtables such as beans, brinjal, pumpkin…....." PD2 |
|  |  | "I usually eat milk, yogurt…...I like then…......I ask to buy for me when I like eat" PD3 |
|  |  | "they don't like okra….because it has slippary substance….they like green leafy vegetables….so I make them what they like" – PD 9 |
|  |  | I don't like vegetable grown in our home garden…we have pumpkin shoots, lufa gourd, pumpkin, green leafy vegetables in home garden…...I don't like pumpkin shoot, lufa gourd and pumpkin" – PDc5 |
|  | Not demanding children | " we some time eat lunch together and some time we don't….in morining if I am out for work then we can't eat together….but we eat dinner together" - PD6  "I have never heard about her dislikes…..she never said she don't like any foods" – PD6  "yes we have green leafy vegetables, green beans, garlic, bean….I like all the vegetables.."- PDc6 |
|  |  | "my children do not get angry…..they eat what I prepare…I do not need to counsel them…." PD7 |
|  |  | "I like all vegetables that are grown in the field…it helps to make our body healthy" – PDc7  "I like most…. potato, green leafy vegetables, bottle gourd, red and black lentils….I like dhido too…..I like stinging nettle the most….I started eating from very small age" PDc7 |
|  |  | "he never refuse to eat. But some time If he is not eating then I show some angry expression then he starts eating…we have only one kitchen and he eats what ever we cook…"- PD8  "yes, we have home garden nearby…. right now we have tomato, cabbage, cauliflower and pumpkin…...yes I like those vegetables…....we have fruits in our shop…...and we have milk in my maternal uncle house…I like milk and yogurt " – PDc8 |
|  | degree of preference | "He has preference, but he never totally disgree to eat" - PD2 |
|  |  | " I prefer all vegetable that are grown in my home except raddish and cauliflower…..but I eat when there is no other option.." - PDC2 |
|  |  | "I like all fruits….. But I don't like some vegetables such as raddish, gralic, cauliflower, squish, bitter gourd…........however I eat them in less amount" – PDC3 |
|  |  | "He don't like to eat bitter gourd….and he prefer less apple but he likes to orange and banana" – PD4 |
|  |  | "I have never heard about her dislikes…..she never said she don't like any foods" PD6 |
|  |  | "I less prefer cauliflower and beans…...."- PD7 |
| Time schedule for food | Eating schedule | "we have fixed time for food…we eat lunch before 10 am….children need to go school around 9 and sir (husband) goes to office…..........we get late in evening because of shop, we eat dinner at around 9" – PD3 |
|  |  | "I usually eat four times: I eat bread and tea in morining, then I eat lunch at around 9:30, after that I eat snacks around 2:30, and finally I eat dinner arund 9" – PDC3 |
|  |  | "we drink tea in the morining….then we go to work, we eat lunch between 10 to 11:30, and finally we dinner before 7 pm" – PD5 |
|  |  | " we cook three times, we drink tea in the morining, lunch at around 10, sancks around 2 pm and dinner around 7-8 pm" – PD6 |
|  |  | "we eat at around 9:00 pm, but children eat at around 7:00 pm and go to bed, they sleep early…." - PD7 |
|  |  | "we eat four times, but my children eat 5 times…...at morining has tea, doughnut/biscuit for breakfast, then lunch at aronud 9:00 to 9:30 am, he eat snack at school at around 1, again he has snack when he comes back from school, and finally at evening we eat lunch" – PD8 |
| Knowledge on diverse food | Parents knowledge on benefit of consuming diverse food | " yes I know the benefit of diverse food…..if we have money, it is good to eat fish, meat, liver, kidney, beans, green leafy vegetables in each food serving…...but we don't have enough money……it benefits both children and us…..it provides required energy"- PD1  "it is better to eat food from our own home garden. Futhermore, didho made from local maize, kodo, rice, fapar etc and vegetable we produce is good for health…." PD1 |
|  |  | "we can get protien from beans…...vitamins from fruits…..vitamin A,B something from leafy vegetables. Similary, I think we can get vitamin, calcium from fish and meat" -PD2 |
|  |  | "when we eat diverse food, it helps to increase immunity power, balance mind of children, and memory power. If we eat fruits, green leafy vegetables in balance, it helps to make our eye sight better. It is very good to eat balanced food" PD3 |
|  |  | "yes diverse food is good for health….....it benefits our body…....we should eat balance peas and beans…...i used to eat in home….In addtion, I came to know from school" -PD4  " it is good to eat some time rice, vegetables, pulses; sometimes we make chapati, dhido…..., some time it is good to cook fish and meat" – PD4 |
|  |  | if we eat balance food then it benefit our digestion system. if we eat monotony diet then it we will develop digestion for particular food…so we should eat diverse food" – PD 5  "if we don’t eat diverse food then It have negative health impact, for example diarrhoe.." – PD5 |
|  |  | "healthy food means those food which benefits our body, for example greenleafy vegetables, peas and beans, eggs, meat and fish…if we eat like this it called healthy food" – PD6  "if there are diverse food it makes healthy…we don't eat regularly..but we try to eat in balance….." -PD6  "if we eat diverse food it gives us energy and if we eat fish and meat then it also provides us growing minerals" PD6 |
|  |  | "diverse food has many good health effect in one way…but on the other had it might of negative impact too…......if we eat legumes, green leafy vegetables and other vegetables…I heard meat has less good effect…." PD 7  "leafy vegetables, green vegetables, legumes…grams, green beans, peas…..benefits our body…...it helps us to have clear eye sight…." - PD7 |
|  |  | "how can I say what makes food healthy…...In used to think food produced locally in village are healthy…nowdays every food are vulnerable, I think every food arre unhealthy…...yes but those cultivated in village without using chemical and fertilizer are healthy.....if we do not put medicine to plant, they die.....so i think vegetables from village are healthy " – PD8  "I think diverse food might be good for health…....if we eat vegetables, meat, milk, yogurt in balance way, then it will benefit health " – PD8 |
|  | Children’s perception on benefit of consuming diverse food | " If we eat food in balance then it helps us to keep healthy…....I learned from school" PDC1 |
|  |  | "…...I know we need to eat diverse food…..it helps to increase vitamin"- PDC2  "healthy food are green leafy vegetables, beans…..." PDC2 |
|  |  | "if we eat diverse food then it provides good health…..it makes our eyes healthy" PDC3  "I my view green vegetables, fruits does good for our body" – PDC3 |
|  |  | "yes I have listened about the benefit of diverse food….it benefits health…it give strength and make our body energetic"- PDc4  "I think food we produce in home is good for health…...."- PDc4 |
|  |  | "if we eat diverse food, it makes helps for eyes" – PDc5  "I don't know about the harm of not eating diverse food.." PDc5 |
|  |  | "if we eat green leafy vegetables it makes eyes healthy…if we eat peas and beans, fish, and meat then it gives energy"- PDc6 |
|  |  | "diverse food benefits our body…. milk provides strength, green leafy vegetables improves eyesight…......" PDc7 |
|  | Effect of monotones diet | " if we don’t eat diverse food, we can get disease ….so to prevent disease ad get strength we should take diverse food" – PD4 |
|  |  | "if we don't eat diverse food then, sometime we can get diarrhea, some time we get sick…we don't feel to work"- PD6 |
|  | Perception of healthy food | "Healthy food are balanced food. If we are able to eat balance food then it benefits our health. To make diet balance we need eat green leafy vegetables un balance…if we eat *dhido* in lunch, then we should eat in chapati in dinner.” – PD5 |
|  |  | "I didn't study benefit of healthy food at school…. (further exploration/ changing question pattern) I think green leafy vegetables benefits us. Meat, milk, yogurt makes us strong…..." – PDc8 |
|  | Children’s perception on healthy food | "in my view…food containing green leafy vegetables, peas and beans, fish, meat, eggs….are healthy" - PDc6  "if we cook food cleanly…..and clean then it makes food healthy"- PDc6 |
|  |  | "healthy food are those which benefit our body…...we should not eat everything…. green leafy vegetables, legumes…..homemade foods are healthy….." – PDc7 |
|  |  | "I don't have much idea about healthy food…...but I have heard that green vegetables are good for health" – PDc9 |
| Knowledge on unhealthy foods | Parents knowledge on unhealthy food | "rotten food, old food are unhealthy, and affects us. Foods from outside are preserved with chemicals, if we eat that food, it will affect our health"- PD1 |
|  |  | "junk food such as noodles, biscuits, chocolates etc can be not good for health" – PD3 |
|  |  | “…..I didn't heard about bad food for health…... but food from outside such as noodles is not good…it errodes our bones.."- PDc4 |
|  |  | "unhealthy food mean oily food, food contaminated with flies, rotten foods etc…." |
|  |  | "unhealthy food are those which affects our health….it depends on our health, for example; if we have high blood pressure then we should eat bitter gourd but if we have low pressure, we should not eat….we don't have much idea…" – PD6  "if there is flies in our food, if we cook without cleaning our hands and feets…it makes food unhealthy " -PD6 |
|  | Children | " food like noodles, biscuit, chocolate and chewing gum is unhealthy food…these food doesnot make our body healthy and strong, it makes our body weak"- PDC1 |
|  |  | "noodles…..... Unfreshed food…..it cause stomach pain, bone erosion"- PDC2 |
|  |  | "if we cook food in unclean way, it makes food unhealthy…. if we don't cook properly then also it makes unhealthy"- PDc6 |
|  |  | "food that’s harms our body are unhealthy foods…. pesticides used vegetables and fruits are unhealthy…that’s what little bit I know…."- PDc7 |
|  | Impact of unhealthy food | "if we eat same food, it is obviousaly unhealthy…....if we eat protein only it is also unhealthy.....if we eat old food it causes diarrhea, vomitting, food poisoning….we need to eat mixed food…....if we eat protien it gives only minerals, minerals are not sufficient for the body..so we need all kind of foods" – PD 3 |
|  |  | "spices contained in noodles causes, bone erosion…..."- PDc9 |
| Source of information | Parents: self + other organizational staff | "I knew about food and nutrition previously…...later people like you gave us more information" – PD1 |
|  | School+ different media | "I got this knowledge while studying…..Now a days, there are some advertisement in Radio/TV for children….there is...eat like this, eat like that kind of information" - PD2 |
|  | From school | "I did nursing…so I know about this…........" – PD3 |
|  | From study and electronic mediums (Radio, TV) | “I knew this by studying and some time we can get information from Radio, TV” – PD5 |
|  | Parents as a source of information | "aunt informed me about this…........and some what I learned form school " PDc3 |
|  |  | "I came to know from school" -PDc2 |
|  |  | "My mom told about the benefit of diverse food" -PD6 |
|  | Own perception | “………..this knowledge is from my own perception, it came from my own mind"- PDc8 |
|  | Teacher as source of information | "My ma'am used to say at school " |
| Snack for children | Food for school snack | "usually eat chapati, soyabean, beaten rice, popcorn in snacks"-PDC1 |
|  |  | "sometime I prepare vegetables and beaten rice, sometime I put fruits for his snacks" - PD2 |
|  |  | "Nowdays, we usually prepare snacks, some time chapati, some time children like noodles, some time beaten rice and eggs, sometime beaten rice and vegetables" – PD3 |
|  |  | "I use to take chapati….some time beaten rice for snacks in school" – PDc4  "When I take money, I used to buy doughnut, sometime vegetables and samosa" |
|  |  | "if available I send snack from home or she come to home and eat " – PD6  " we send snack from home… some time we send maize and soyabean, some time fried rice….some time they want to eat biscuit…...." – PD6  "I don't get money for snacks" – PDc6 |
|  |  | "some time they prefer fried potato, some time they like milk and biscuits, and some time I make jaulo (mixture of rice, lentil, and vegetables)…...."- PD7  "For those who study nearer school, I prepare them rice, pulse and vegetables. But for those who study far, I prepare them tiffin…..some time I prepare bitten rice, biscuit with milk….they eat during their interval" – PD7  "Yeah in addition to snacks…some time they ask for money, I give them money even it is less…I give them 5 rupees…..so that they can buy chocolates...they feel like eating when they see other children buying"- PD7  "sometime eat biscuits, some time eat thupka, and sometime I take snacks from home" PDc7 |
|  |  | "school provides snacks, but send from here also, because I think school snacks is not enough…..he eats again when he come back from school ." – PD8  "some time I send fried potato…he don't want to take other vegetables…..some he eat buscuits, sphagatie, pakora…and some time he takes noodles." – PD8  "I take pop corn, some time biscuits, some time mo:mo…......" - PDc8  "yes some time I ask money for snack, and I eat vegetables and *malpuwa* (a kind of snack made up of flour), *thupka* (spagattee with soup)…....I prefer eating mo:mo for snack"- PDc8 |
|  |  | "I send them rice for snack at school…....I used to buy them tiffen, but they usually lost….so I take rice to their school" – PD9 |
| Snack preference | Homemade snack | "my friends bring money for snacks and buy noodles….....I also do some time..but I prefer chapati… I can eat with tea and vegetables also" PDC1 |
|  | Preference of different snacks | "children don't prefer to eat same snacks everytime…....sometime they eat bread…some time beaten rice, some time chapati"- PD3 |
|  |  | "I like to eat potato and chapati for snack" – PDc3 |
|  |  | "I love eating rice/milk porridge in snacks"- PDc6 |
|  |  | "yes some time I ask money for snack, and I eat vegetables and *malpuwa* (a kind of snack made up of flour), thupka (spagattee with soup)…....I prefer eating mo:mo for snack" PD 8 |
| Counseling/nudging to eat | Adding food that children likes | He don't like beans, I counsel him to eat beans…...I must add potato with beans…..he wants less beans" PD2  "when I say its good to eat to him…....he eat…he has some degree of preference only” -PD2 |
|  |  | "I don't give food as a prize…...he is reluctant to food, he eats what he want….he don't eat all vegetbales. He eats when there is vegetables made in their way, pulsees…., it might be due to his sickness- PD5  "there are chldren who don't eat eveything. Our child is also like that, he don't like pumpkin, bottle gourd. In this situation we usually prepare what he likes. Some time he want to eat noodles, we buy that and counsel him to eat food" - PD5 |
|  |  | "he less prefer apple, but when I cut and give to hime he eats…he eats all vegetables those he preferes, but if he don't like he leave piece, for instance, capsicum, chayote squash…......but it doesnot mean we need to prepare other foods, if we prepare lentils it works for him.........In that case, I some time I mix those vegetables with other vegetables such as meat, pumkin...he eats when I mix...." PD8 |
|  | Counseling as per the mood | "Yeah! We need to counsel them some time…some time we need to threaten them to make them eat…...we need to see the mood of children” – PD3  "when we show too much love its not good…and its not good to show anger every time…..we need to balance every time"  "yes! If I counsel her she eat, but I should cook little bit in good taste"- PD3 |
|  | Advice provided to children | "when I give money to children, some time I advise to eat eggs…..we bring eggs to home also"- PD4  "eventhough he prefers less bitter gourd…....he eats when I cook at home…." – PD4 |
|  | Food reward | "Yeah, sometimes I make chapati in oil, sometime noodles with eggs…for making them happy" – PD4 |
|  | Parents showing gesture | "he never refuse to eat. But some time If he is not eating then I show some angry expression then he starts eating…we have only one kitchen and he eats what ever we cook…" PD8 |
| Culture | Change in culture | "Nowdays there are no people like past….Now there is drastic change in village also…. In past people didn't care about children… they don't know what is good to feed their chidren….Now even in village has changed, I think this is because of the informtion they heard" - PD2 |
|  |  | "Now there is no such social belief, there migh have in past. People have changed now. But, we are chettri, still we do not eat meat of pig, buffalo in home. However, outside of home people eat. We also eat out side of home" - PD2 |
|  | Ethnicity difference | "Now there is no such social belief, there migh have in past. People have changed now. But, we are chettri, still we do not eat meat of pig, buffalo in home. However, outside of home people eat. We also eat out side of home" – PD2 |
|  | Ethnicity | "we don't have such kind of any food restriction, we are *Newar* caste, that why we do not have any specific things to not to eat" PD3 |
|  | Restriction during pregnancy only | "In past, pregnant women were advised not to eat much food…. It was thought that if pregnant mother eat lot, then it results childs suffocation. Nowadays, I don't have such idea"- PD1 |
|  | No any restriction | "we don't have any cultural restriction to food…....in past pregnant mothers were restricted to eat green vegetbles….." |
|  | Belief | "If we have chest pain, throat pain, then we eat dhido and leafy vegetables… people thinks it helps to get relief" – PD8 |
| Freedom for choosing food | Express their preference to their prents | "I ask her to cook food when I feel to eat….I like soyabean, pulse…" - PDC2 |
|  |  | "yes, they prepare my preferred food, when I ask them…....." PDc4 |
|  |  | "yes I ask my mother to cook what I like…...I like bitter gourd, Soyabean…" – PDc5  "yes, I can get what I like….I like bitter gourd" – PDc5 |
|  |  | "Yes I say if I want to eat any food… she cooks if available..." PDc6  "I can choose what I eat…..........but I don't have any dislike" PDc6 |
|  |  | "yes! We can ask what we like to eat to mom…..” PDc7 |
|  |  | "Yes I can request what I prefer…and my mom cooks for us" – PDc8 |
|  | Cooking according to children’s preference | "My children cook their self what they like….for younger one I cook if she want to eat anything "- PD9  "they don't like okra….because it has slippary substance….they like green leafy vegetables….so I make them what they like"- PD9 |
| Decision | Children decision | "I cook whatever I like to eat…..."- PDC1 |
|  | Participatory decision | "some time mom says what to cook….and some time we decide what to cook…." PDc7 |
|  | Mom’s decision | "I eat what mommy cook…she cook meat usually on Saturday"- PDC2 |
|  |  | "aunty decide what to cook and eat in our home" – PDC3 |
|  |  | "my mom decide what to eat today" – PDc5 |
|  |  | "Whatever mom decide we eat…." - PDc8 |
|  | Father ‘s decision | "my father decide what to cook" – PDc4 |
|  |  | "my father decide what to eat" – PDc9 |
| Frequency of meal | Consumes four times a day | "I eat three time….breakfast, snack, and dinner"- PDc1 |
|  |  | "we usually eat four times a day….day lunch, evening dinner, morning breakfast and day snack" |
|  |  | "we eat chapati and beatan rice in breakfast, then we eat rice in lunch…..and some time snack… and dinner" – PD4  "I usually eat tea, biscuits, chapati in breakfast…...I usually eat 4 times a day " – PDc4 |
|  |  | "we eat three times….in addition morning we have tea with doughnut, puff…....... Then we eat lunch…at day time, we prepare snacks on their demands….in total we eat 3-4 times in day" – PD7 |
|  |  | "we eat four times, but my children eat 5 times…...at morining has tea, doughnut/biscuit for breakfast, then lunch at aronud 9:00 to 9:30 am, he eat snack at school at around 1, again he has snack when he comes back from school, and finally at evening we eat lunch" – PD8 |
| Perception on not consuming diverse food | Money is not only the reason | "Not eating due to money is not an issue here…...those who can buy food, or those who cannot buy, can get work easily...but I don't know about the more rural areas…....here we can easily get work"- PD3  "if you feel lazy, you don't go for work, it is different scenario…otherwise you can get work and can get 1000 Rs per day, which is nice income"- PD3 |
|  | greediness | "the main reason behind this is due to greedy nature of people. Rich people have greedy for their wealth, but poor thinks to eat for the and can manage even by searching" – PD5 |
|  | Willingness and laziness | "I feel if we have enthusiasm and wish, it is possible to get diverse food….....if you are not lazy , you can produce in home and you don't need to buy" – PD6 |
|  | Willingness | "we live by day to day income generating….we should eat unless we have last breath…..we don't want to save lot…..we eat what we have and we have to make our every member happy….if we do not eat we will get thin…....we want to live happy with good foods......we feel lets eat today, for tomorro will generate income again........”- PD7 |
|  |  | "nowadays everyone eats diverse food….if there is no money we can work and get money…..." – PD8 |

**Compiled themes generated from NDs group interview (Raw)**

| **Themes** | **Sub-theme** | **Quotes** |
| --- | --- | --- |
| Participatory cooking/ Involving children in cooking | Grandmother and mother | "usually mother and sister in-law prepares food.."- ND1 |
|  | grandmother and mom | "Mother (mother in-law) cooked food…...but usually I cook food"- ND3  "……my *aama* (grandmother) made food today ……..usually my mom cooks food" – NDc3 |
|  | Mother | "my mother cooks every day…...yes I ask my mother if I want to eat anything" – NDc1 |
|  |  | "we ate food around 7 pm. I cooked food, because my wife is sick…usually my wife cook food" – ND5  "yesterday my father cooked food….usually my mother cooks food" – NDc5 |
|  |  | "Usually my mother cooks food" – NDc7 |
|  |  | "I cooked sir…..usually I cook in my home…some time children cook, when they do not have school…" – ND7 |
|  |  | "yes we ate lunch at around 10 am….I cooked chayote squash and green leafy vegetables…...(rice is complementary)” – ND8  "usually I cook myself…."- ND8 |
|  | Male involvement | "I cooked myself…...I cooked bottle gourd, potato and luffa gourd together…" – ND4  "My dad and mom both cook food " – NDc4 |
|  | Grandmother | "I cooked my self…...."- N9  "usually my daughter in-law prepares food…."- N9  "my grandmother made food…......" – NDc9 |
|  | Children involvement | "my daughter cooked…......she made potato, pumpkin shoot and soyabean" – ND2  "Usually I cook food …..... " – ND2  "some time me and some time mom cooks food…" – NDc2 |
|  |  | "we both cook food.....she cooked rice and other I cooked myself….when I go out she cook" – ND6  "Usually my mom make foods…" – NDc6 |
|  | Less time for cooking | "some time we cannot have time to cook…...if it is late in evening, for fast we make fermented vegetables with soyabean…...thats all what I think" – ND1 |
| Availability/Accessibility for the different food groups | Home production | "we only produce soyabean…....yes, we have home garden and we have just cultivated green leafy vegetables, then we have beans…..."- ND1  "In one year we cultivate potato…..after harvesting paddy we cultivate green leafy vegetables, potato, beans….some time we cultivate black gram pulse for eating…and some time we cultivate peas but it does not grow weell, due to lack of irrigation water.."-ND1  "we don't have issue of food…...we can get when we want to eat" – ND1  yes, we have home garden…..we have cucumber, pumpkin, green vegetables and chayote squash" – NDc1  "throughout the year we grow green leafy vegetables, chayote squash, pumpkin…" – NDc1 |
|  |  | "we produce vegetables such as, green leafy vegetables, luffa gourd, brinjal…....we also produce rice, maize, peas…....." ND2  "we have vegetables but we don't have fruits…....." – NDc2  "yes we have milk and yogurt…..I like it….. " -NDc2 |
|  |  | "we don't produce all that we cook. We cultivate green leafy vegetables, pumpkin, potato…..we buy pulses…..we produce cereals such as maize, millet.."- ND3  "we don't have milk/yogurt…, and, fish and meat"- ND3 |
|  |  | "I don't have to buy rice, peas and beans, and vegetables from market…I am doing farming on rental land….agriculture is my major occupation….." – ND4  "I have 10 goats, few local hens and buffalo….........I used to work in Malaysia before, I found very hard to work there. So. I came back to work in my own village…..... I have rented small land for farming " – ND4  "we eat vegetable and pulse. Pulses work as legumes, and we cook little bit vegetables. Nowadays we have lot of bottle gourd, luffa gourd, pumpkin…...…...we have leaf mustard as green vegetables..80% people in this village are farmer and we sell our vegetables" – ND4 |
|  |  | "I don't want to lie. We produce vegetable in home, such as: green leafy vegetables, radish….Other food we need to buy. We don't have paddy cultivation, so we need to buy rice. Among flour, we have millet and dhido" – ND5  "we don't have irrigation facility now, so we do not cultivate wheat"- ND5  "we don't have milk production. In this village we do not rare cattle for milk…few of them rare goat" – ND5  "I don't have better knowledge on healthy foods…..I am not well educated, so I don't know much…...we only eat what we produce in each season" – ND5  "we have green leafy vegetables in home garden….but I don't know what kind of vegetables…..recently I have not been to my home garden" – ND5 |
|  |  | "food we produce is not enough….we need to buy.." – ND6  "I don't have cultivated much now….just pumpkin shoot, pumpkin, radish and mustard"- ND6  "I have only green vegetables but I buy little bit potato, pulses, rice….. from market.. we don't have legume production except peas in some season"- ND6 |
|  |  | "currently we have luffa gourd, green leafy vegetables, green beans, radish, okra, and chilly"- ND7  "I have hen in home, and I have goat too" – ND7  "we rear cattle too….two to four month we can have milk too" – ND7  "we have green leafy vegetables, luffa gourd, green bean, beans, chayote squash, okra…...." – NDc7 |
|  |  | "we don't have lentils but we produce vegetables…......we produce paddy, maize, millet, peas…...and we have goat"- ND8  "currently we have leafy vegetable, chilly, little bit pea, radish, coriander, and little bit of radish and cucumber" – ND8  "we do not have any frutis…" – ND8  "we have radish, leafy vegetables, garlic, onion, cauliflower and cabbage in our home garden" – NDc8  "we have radish, leafy vegetables, garlic, onion, cauliflower and cabbage in our home garden"- NDc8 |
|  |  | " …we produce millet, maize and paddy…...we only cultivate green leafy vegetables"- ND9  "I have hen for meat, but it takes time to grow…..we have availability of milk and yogurt in our house" – ND9  "we have green leafy vegetables, garlic, potato and tomato…..I like all the vegetables" – NDc9 |
|  | Buying from the market | "we buy rice, we don't need to buy vegetables…..but we need to buy peas and beans”- ND1  "we need to buy pulses, peas and beans, and fruits from the market" – NDc1 |
|  |  | "we don't have red pulse, meat…..so we buy from the market" – ND2  “……..but we need to buy eggs, fish and meat”- ND3 |
|  |  | "we don't buy much from the market, but we should buy oil, spices, lentils, legumes…..this snail don't allow to grow legumes” – ND4 |
|  |  | "I don't want to lie. We produce vegetable in home, such as: green leafy vegetables, radish….Other food we need to buy. We don't have paddy cultivation, so we need to buy rice. Among flour, we have millet and *dhido*" – ND5  "we need to buy other food except vegetables" – ND5 |
|  |  | "we have home garden….we produce in our home…..mostly we buy lentils from the market…… in some season we buy potato.."- ND7 |
|  |  | "we buy lentils, potato, beans…."- ND8 |
|  |  | "we need to most of things from market….rice, oil, spices, potato, cabbage, vegetables…..I have only cultivated green leafy vegetables" – ND9 |
|  | Far market | "some time I like eating junk food when I go to markets…..market is little bit far from our home."- NDc2 |
| Sharing of food | No sharing | "we don't share produced food…we eat if we produce. Otherwise, we buy…..." – ND3 |
|  | Sharing with neighbor | "we usually share vegetables and fruits in our community…...." – ND5 |
|  |  | "Yes! We share vegetables if other do not have, and they also share their vegetables" – ND7 |
| Food consuming behavior | Current consumption | "we ate pulse, rice, potato with green leafy vegetables…......" – ND1  "yes I ate snacks.. I ate maize and tea…."- NDc1 |
|  |  | "yes, I ate pumpkin shoot, potato and soyabean, milk, and rice" – NDc2 |
|  |  | "we can say we cook one type of vegetables such as green leafy vegetables, some time we mix vegetables potato and green beans"- ND3  "we eat dhido in the morning because my husband has diabetes but we make rice in dinner"- ND3 |
|  |  | "I ate green beans, rice and meat….my *aama* (grandmother made food" – NDc3 |
|  |  | "I ate luffa gourd, gram, dhido and rice" – NDc4 |
|  |  | "we ate chayote squash potato and rice.."- ND5 |
|  |  | "we cooked rice, pulse and meat" – ND6 |
|  |  | "its festival time so we ate dry meat, pulse and rice…...potato, green bean, lufa gourd mix vegetables.." – ND7  "yes! I ate *khana* (lunch/dinner)…..we had dry meat, rice, green beans…." – NDc7 |
|  |  | "yes we ate lunch at around 10 am….I cooked chayote squash and green leafy vegetables…...(rice is complementary)" |
|  |  | "yes we ate lunch…we ate rice, chayote squash and potato….." – ND9  "I ate chyote squash, rice, potato and soyabean" – NDc9 |
|  | Usual consumption | "we eat green leafy vegetables in lunch…...for evening if we have peas and beans, we add potato and make gravy and then we eat…some time we eat lentils" – ND1  "we eat different food, sometime peas and beans, and some time potato mixed with fermented vegetables…....For meat, sometime we eat once a week or once a two week"- NDc1  "we don't eat green leafy vegetables, peas and beans regularly…."- NDc1  "we have vegetables….but we don't like to eat regularly " – NDc1 |
|  |  | "we usually make pulse and vegetables…some time we make only one type of foods…....usually our *khana* (lunch and dinner) is same but we make different snacks…." – ND2 |
|  |  | "we generally eat rice in both lunch and dinner, some time we eat half rice and half *dhido* in dinner, and in snack we eat chapati ….." – ND4  "we use food that is cooked in the morning….we keep it safely and eat in evening, and we heat it before eating…...but we do not eat food that is cooked at evening, we give those food to animals "- ND4 |
|  |  | "we eat rice and pulses regularly…..we cook vegetables in alternate way….sometime fish/meat, sometime eggs, and sometime green vegetables" – ND6  "I eat late in the morning…...if I ate rice, pulses, and vegetables in morning , then in evening I make fermented vegetables…........hmmmmm" |
|  |  | "we eat different vegetables in morning and evening…if we eat legumes in morining…...then we will prepare chayote squash and leafy vegetables in evening….." – ND8 |
|  | Only reliable on what produced | "some time we eat green leafy vegetbales, lufa gourd, some time pulses…it depends upon production " – ND5  "we usually eat rice at both lunch and dinner….." – ND5 |
| Parents behavior | Parents behavior regarding junk food | " we should not eat much packed food, but we can eat when needed, not much but in balanced way in balanced time….... "- ND1  "we eat those junk food 4-5 time a month" – ND1 |
| Eating together | Taste good | " we all eat together with children…if we eat together, distasteful food also become tasty " |
|  | Lunch separately during school days | "we first give food to those who will go school, after that we eat "- ND3 |
|  |  | "we eat lunch separately during our school days. But we eat dinner together" – NDc4 |
|  |  | " we eat together….........." – ND5  "During school days I eat bit earlier than my parents…...." – NDc5 |
|  |  | "we usually eat together, during school days children eats little before than us.." – ND8 |
|  | Feels lonely/ feels good | "it good eat together….I don't feel to eat alone…so we eat together.." – NDc6 |
|  |  | "we eat together sir…we like eating together" – NDc7  "Yes we eat together…" – NDc7 |
|  |  | "we eat food together…...I think it's good to eat together"- ND9 |
|  |  | "we eat together, because I feel good when I eat together with my family"- NDc2 |
| Parents preference | Prefer to consuming mix vegetable | "we preferer eating mix vegetables, we do not like eating only one kind of vegetables" – ND7 |
| Children preference | Preference of specific food | "I don't like to okra….I feel not to eat because it is slippery" – NDc1 |
|  |  | "yes, I like green vegetables, green beans, pumkin…..I don't like lufa gourd….and among fruits I don't like banana"- NDc3  "our housholehold do not have milk and yogurt, fish, eggs and meat…but I like it”- NDc3 |
|  |  | "I don’t like eating bitter gourd…other I can eat…...” – NDc6  "some time I get craving, so I eat fastfood" – NDc6 |
|  |  | "I don't like lufa gourd….and I less prefer green leafy vegetables " – NDc7 |
|  |  | "except cabbage, I like all the vegetables grown at home garden" – NDc8 |
|  | Don’t like eggs | "yes, we have milk and I like it"- NDc9  "Yes, we have hen for meat for I don't like eggs."- ND c9 |
|  | Demanding money | "actually children don't want food as incentive, they want money. If we give money, they usually buy noodles and biscuits…" – ND3  "I give money for lunch…...yeah! When I ask what did you eat, he says like chatpat, panipuri, chewingum, chocolate…......" – ND3 |
|  | Not demanding children | "They don't say no to cooked food sir….they eat all if I cook…" – ND8 |
|  |  |  |
| Time for food | Eating schedule | "we usually eat four times, 2 times khana (lunch and dinner) and 2 time snacks…..we eat breakfast around 7 am, lunch at 10 am, snacks at 2 pm, and dinner at around 7-8 pm."- ND2 |
|  |  | "children usually eat lunch first around 8:30 - 8:45, then we eat around 10 am, because at morning one of us is busy with dealing with household cattle and one serves food for children. But we eat dinner together" – ND4 |
|  |  | "we eat lunch around 10 am, then tea and snacks around 2 to 2:30 pm, and finally we eat dinner at around 8:30 to 9:00 pm…in winter we eat little early…..." – ND8 |
| Knowledge on diverse food | Knowledge on diverse food  Benefits of diverse food | "Actually, green leafy vegetables make eye healthy. Peas and beans also same …we can get little bit vitamin, but I don't know much…it would be better if you could tell me…........I don't know what happens when we eat same food" – ND1  "I don't have listen about the benefits much….I know we should not eat old food…..we should wash vegetables before cutting, and once we cut we should not wash it again…...it is same for potato …......this what I heard and do the same"- ND1  "if we eat diverse food it makes taste different…....we cannot add to potato to all”.- ND1  "if we eat only one type of food it may be not good for taste, it might not do good for health… I don't know much about the health impact"- ND1 |
|  |  | "We were informed that green leafy vegetables are good for eye sight….home made foods are better for health. It prevents us from being diseased, and also increased our immunity power" – ND4 |
|  |  | "if we eat diverse food it makes our body active…" – ND3 |
|  |  | "if we consume diverse food then it will benefit our body…… consuming only one same food might not benefit, so it better to change food" – ND5  "I think eating vegetables and fruits benefit our body…..I think it provides vitamin C and similar kind of vitamins… I don't know much" – ND5 |
|  |  | "if we eat fish, meat, fruits, then they provide us vitamin. It helps to improve immunity power" – ND6 |
|  |  | "I think healthy food means, if we eat mixed legumes, yogurt, milk…....I think so.."- ND7  "fruits, milk, yogurt, legumes etc makes food healthy…" – ND7 |
|  |  | "if we eat green leafy vegetables it helps our eye's vision….we can get vitamin…if we eat yellow fruits, and , peas and beans it provides nutrients"- ND2 |
|  |  | "if we eat diverse food, sir said it helps to improve brain….grow our body…I don't know much…"- ND7 |
|  | Effect of monotones diet | "if we don't eat diverse food then it can cause night lightness…and can cause other disease"- ND2 |
|  |  | "I know if diet is monotonous it is not good for health but I don't know what it is…."- ND3 |
|  |  | "if we don't eat diverse food, it is not good…..it may cause disease…..only one food is not good" – ND6 |
|  |  | "I heard that if we do not eat diverse food it may cause disease ….....so it’s better to cultivate in home" – ND7 |
|  | Healthy food | "we eat green leafy vegetables and potato twice a week, gram twice a week…..we should soak gram one day before eating, we do the same"- ND1 |
|  |  | "green leafy vegetables, peas and beans, eggs makes food healthy” – ND3 |
|  |  | "healthy food means if we cook today and we use tomorrow, then it is unhealthy food….old food is not healthy….For healthy food, utensil to every thing should be clean…." – ND4 |
|  |  | "healthy food contains pulses, rice, vegetables, green leafy vegetables, eggs, fish, meat and fruits…..." – ND6  "we need healthy food for satisfying our hunger….next it helps to keep us healthy" – ND6 |
|  |  | "I think fish and meat are nutritious food" – ND8 |
|  |  | "I heard that , we must consume legumes and green leafy vegetables, but I don't know"- ND9 |
|  | Children perception on benefit of consuming diverse food | "I don't have idea about eating diverse healthy food…..but teacher says us to eat balance food…...I think, it has benefit for our body…....if we eat such food it does not hurt our stomach"- NDc1 |
|  |  | "diverse food makes our body active…."- NDc2 |
|  |  | "yes I have heard about the eating diverse food…...it gives energy and keeps healthy" |
|  |  | "Diverse food is good for our body…it makes us healthy" – NDc8 |
|  | Effect of consuming monotone food | "if we eat same food regularly then the taste of that food become distasteful" – NDc1 |
|  |  | "if we don't eat diverse food then it can makes us physically disable" – NDc2 |
|  |  | "if we eat monotones food then it weakens our body…." – NDc6 |
|  | Knowledge of healthy food | "I know which food are good for health….green leafy vegetables, peas and beans " – NDc1 |
|  |  | "I think healthy food are green leafy vegetables, peas and beans, yellow fruits etc.…....if we cook them clean then its healthy food" – NDc2 |
|  |  | "green leafy vegetables, peas/beans…are healthy food….." – NDc3 |
|  |  | "healthy foods means eating clean, eating fresh and eating in clean utensils" – NDc4 |
|  |  | "yogurt, milk, fish and meat, eggs and fruits are healthy foods…...green leafy vegetables" – NDc6  " if we healthy food, it helps to give energy to our body …if we eat green leafy vegetables, it makes our eye healthy…...it also helps to prevent malnutrition and prevent our intestine from wasting" – NDc6 |
|  |  | "green leafy vegetables, legumes, fish and meat are healthy food.." – NDc7  "I eat healthy food because I don't get disease" – NDc7 |
|  |  | "Yes I know about healthy food….green leafy vegetables, legumes are known as healthy food…......"- NDc9 |
|  | Forgot | "I used to know about healthy food…but I forgot now…....”- NDc5  "if we eat green leafy vegetables then it is healthy…..I knew from school" – NDc5 |
|  |  | "I don't know about benefit of diverse food sir…..(after remembering) yes, one time teacher taught about this in school but I forgot…" – ND8 |
| Knowledge on unhealthy foods | Parents concept of unhealthy food  Unhealthy food  Unhealthy food | "I don't have knowledge about the unhealthy foods….....but if we eat lot of noodle then it erodes bone….if we eat from shops then it is old and unhealthy"- ND1  "we didn't get information from other medium, we got information while our study…....we learned that hard food are not good for digestion and health"- ND1 |
|  |  | "un healthy food are those which are old and rotten…..it has negative impact on our health…......if food are dirty and files are seen in food, it makes food unhealthy " – ND3 |
|  |  | "I think most of food available in market are unhealthy, such as, chocolate, chemical used bread......they affect our health…..those kind of food I think are unhealthy food" – ND4  "unhealthy foods are date expired food. If we preserve food carelessly at home, then also it is unhealthy. If we buy rotten food, food with chemical, and expired fruits are unhealthy food. If we prepare food in unclean utensil then also it will be unhealthy food" – ND4 |
|  |  | "Unhealthy food are those which harm our body…I think noodles and biscuits… Teacher has asked not to give children…...some people coming from different organization also asked us not to give noodles…...." – ND7  "eventhough these foods are nor good for health….children some times demand this, they don't want to other things" – ND7 |
|  | Don’t know | "I don't know about unhealthy food…." – ND4 |
|  |  | "I don't know what makes the healthy foods… and I have no idea about unhealthy food "- ND9 |
|  |  | "I don't know about unhealthy foods and diverse food.........I haven't read that" – NDc9 |
|  | Children concept on unhealthy food  Unhealthy food  Unhealthy food | "I think alcohol are unhealthy foods " – NDc1 |
|  |  | "biscuits, noodles, *kurmure*, these are unhealthy foods" – NDc2  "some time I like eating when I go to markets…..market is little bit far from our home."- NDc2 |
|  |  | "rotten and old food are unhealthy food…"- NDc3 |
|  |  | "rotten food , unclean food , noodles, biscuits, dalmoth are unhealthy food" – NDc4 |
|  |  | "I don't know about unhealthy foods…...but heard that if we eat noodles lot it errodes our bone " – NDc5 |
|  |  | "I think…...if we eat food without cleaning, and if we eat same food every day, it is unhealthy food….." – NDc5 |
|  |  | "I don't know what makes food unhealthy" – NDC7  "I noodles, *biscuits* and cholates are unhealthy foods…...I eat some time…because they taste good " – NDc7 |
|  | Impact of unhealthy food | "if we do not eat healthy food.. It may cause wasting, malnutrition disease…...there are many other disease" – ND6 |
|  |  | "I think sour food (*panipuri*), dry food (bitten rice, noodles ) affects our health…." – ND8 |
|  |  | "noodles, biscuits, *panipuri*, *chatpate*..are unhealthy food" – NDc8 |
| Source of information | Information from radio and meeting | "I knew about food and nutrition from F.M (radio)…by reading myself and sometime while attending meetings…" – ND2 |
|  | Study course and teacher | "I knew about diverse food from reading and teacher taught us …" |
|  | From school | "I got information from surroundings knowledgeable people, school, and from radio also" – ND3 |
|  |  | "teacher conducts meeting once a month and invite us…...." – ND7 |
|  | From meeting held by different organization | "I knew this information from meeting in school. In addition, people from different organization call for meeting at school. We do have female health community volunteer; she some time provide us information on how to feed children"- ND4 |
|  | TV/ radio/FM | "I got information from TV advertisement, radio, FM..........." – ND5 |
|  | Teacher and meeting | "I had some basic idea from beginning, then after the school and home garden project I kew more about the food and nutrition…..I attend meeting in school and teacher gives us information" |
|  | Children from school | "I got information about healthy food from study…."- NDc3 |
|  |  | "I got information about diverse food from my teacher…....and some time my parent also mention about it" – ND4 |
|  |  | "if we eat green leafy vegetables then it is healthy…..I knew from school" – NDc5 |
|  |  | "I got to know about diverse food from my school" – NDc6 |
|  |  | "I got information about the food and nutrition from the school "NDc7 |
|  | Teacher as source of information | "I had some basic idea from beginning, then after the school and home garden project I kew more about the food and nutrition…..I attend meeting in school and teacher gives us information" |
| Snack for children | Day snack | "we eat 2 -3 three times a day…..... some time I eat noodles and some time I eat rice …......"- NDc1  "I eat noodles and some time gram and eggs in school…" – NDc1 |
|  | Perception on snack they consume | "we feel every thing is healthy…..i eat those bought food 1-2 time a week.." – NDc1  "my friend also eat similar kind of snacks…......some time we eat chapati and thupka too" – NDc1 |
|  | Preparation for school snack | "we prepare snacks for children. Some time we make fry beaten rice and egg, some time maize, sometime rice and pulses ….." – ND2 |
|  |  | "I take maize, chapati, egg and beaten rice, cooked noodle for my snack" – NDc2 |
|  |  | "I take roasted beaten rice, egg, maize and soyabean, chapati made up of millet….." ND4 |
|  |  | "I send children with snack. Teacher ask us to send cooked snack with children. Some time I send them egg and bitten rice, some time I send friend rice, and some time maize and soyabean"- ND7 |
|  |  | "I take rice, sometime bitten rice and eggs, sometime corn and soyabean…for snack" – NDc7  "some time I get money and I eat *thuppa* and samosa in canteen"- NDc7 |
|  |  | "school is nearby so they come back to home and eat snack…......some time they eat chapati and sometime spaghetti…I have small shop…...."- ND8 |
|  |  | "I take bitten rice and *dalmoth*, biscuits…....my friends also brings same things…...I prefer biscuit, because I like taste of biscuits…...dad and gives me money for snacks and I buy biscuits" – NDc9 |
|  | Not taking snack in summer | "we send them maize and soyaben in winter, but during summer they don't want to carry snacks. Some time I give them money and some time we ask to share some food between children" – ND4 |
|  | Money for snack | "School is farfrom home they have to walk for 1 hour.........they have to walk there is no transportation…. we have rules that children should study in their ward school, so they have to walk for long time. We have school near, but they belong to another rural municipality. they have to carry snack long way, so if I ask them to take snack they refuse and I give them money...and if they get money they buy junk food from the market as their wish "- ND4 |
|  |  | "Nowadays we give money for snacks….....mostly children eat noodles, *chatpate*….....they ask money and we give them money….." – ND5  "I take money for snacks…I buy noodles, biscuits, *chatpate*…....." – NDc5  "If we eat much it will not benefit…...but I feel like eating"- NDc5  "My friends also bring money for snack…......" – NDc5 |
|  |  | "I generally do not buy noodles for children, but I give biscuits with tea for breakfast….." – NDc6  "at school we eat…porridege (*jaulo)*, maize and soyabean, fried rice, chapati, bitten rice…......" – NDc6 |
| Snack preference | Homemade snack preference | "I like maize and soyabean for snack…...because they are healthy" – NDc2 |
|  |  | "I prefer eating milk and beaten rice for snacks…..at school I prefer chapati" – NDc4  "In summer days I take money for snacks….because its heavy to carry" – NDc4 |
|  |  | "I prefer fried bitten rice for snack" – NDc7 |
|  | Money | "I usually take money for snacks, and I eat *chatpat*, *panipuri,* sometime eat chapati vegetables also …..my friend brings rice in school for snack" – NDc3  "I prefer eating meat and beaten rice…...some time we can in school" |
| Counseling/nudging to eat | No food reward | "No we don't give food as prize to your children"- ND1 |
|  |  | "No I don't give any food prize for my children" – ND2 |
|  | Food reward | "yes they my family provide me *chamre* (fried rice)…some time dry food such as noodles….to make me happy..."- NDc1 |
|  |  | "if chldren do good in exam I make cook eggs for them…" – ND4 |
|  |  | "my parents cook eggs or meat to make us happy " – NDc4 |
|  | Present for children | "my parents some time brings noodles, biscuits, chocolates….........." – NDc5 |
|  |  | "yes I give buscuits and noodles to make children happy"- ND9 |
|  |  |  |
| Culture | No culture that hinders | "we don't have any culture that stops us to eat food…..we have restriction in case of any die.."- ND1 |
|  |  | "we are Majhi community, we relatively eat more fish and meat… some time we bring legumes….....we celebrate dashain, if someone dies we perform rituals during dashain time…we perform rituals and then only we proceed for other things…." – ND5 |
|  |  | "No! we do not have any kind of culture that hinders us to eat different food "- ND6 |
|  | Ethnicity | "we are Newar, we have many festivals. Even in Dashain and Tihar, we make 10-12 food items of legumes……we around 15 house of Newar…we all celebrate same culture..." – ND7 |
| Freedom for choosing food | Express preference to parents | "yes I can get what I like to eat" – NDc1 |
|  |  | "yes I can ask my mother what I like, and she prepare for me"- NDc2 |
|  |  | "yes, I can express my preference, but we don't have many things here…...." NDc3 |
|  |  | "I can request my preference to my parents…..they cook for me…" - NDc4 |
|  |  | "Yes I can ask if I want to eat anything…..they cook for me" – NDc5 |
|  |  | "If I like to eat anything, I can ask to my mother…..she cooks for me.." – NDc6 |
|  |  | "yes, I can share my preference to my mother….and she cook for us…...."- NDc7 |
|  |  | "yes I share my preference to my mother and she cook for me.." – NDc9 |
| Decision | Family member | "our family member decides what to eat and not to eat…..."- NDc1 |
|  | Grandmother decision | "My *aama* (grandmother) makes decision about what to cook" – NDc3 |
|  | Mom decision | "my mother decides what to cook…...."- NDc2 |
|  |  | "My mother cook food…and we eat….my mother decide for the food" – NDc5 |
|  |  | "My mother decides what to cook…....."NDc6 |
|  |  | "my mother decide what to cook"- NDc7 |
|  |  | "my mother decides what to eat" – NDc9 |
| Frequency of meal | Four meal | "we usually eat food four times a day…..breakfast at morning, then lunch at around 9 am, snack around 3 pm, and evening lunch is not fixed" – ND1 |
|  |  | "we eat 4 meal, 2 times *khana* (lunch and dinner) and 2 times snacks"- NDc2 |
|  |  | we eat four times a day…morning breakfast, then lunch, snack at day, and dinner in evening,…....some time we only eat rice once" – ND3 |
|  |  | "We eat about four meal…......" – ND4 |
|  |  | "we drink tea in the morning, then we eat lunch. If we are at home in day time, then we eat snack. Otherwise we do not eat snack. Finally we eat dinner at around 7 pm" – ND5 |
|  |  | "we eat four meal a day.....I prepare milk and eggs for breakfast, at day I make *jaulo*, soyabean…..etc" – ND6 |
|  |  | "we eat four meals, two times snacks and two times *khana* " – NDc7 |
|  |  | "we eat lunch around 10 am, then tea and snacks around 2 to 2:30 pm, and finally we eat dinner at around 8:30 to 9:00 pm…in winter we eat little early…..."- ND8 |
|  |  | "If we make snack…., then I prepare food three times…..including snacks we eat three time a day…some time we do not eat snack…... " – ND9 |
| Perception on not consuming diverse food | Less time | "some time we cannot have time to cook…...if it is late in evening, for fast we make fermented vegetables with soyabean…...thats all what I think" – ND1  "yes time….everyone works and come late with tiredness, we can boil fermented vegetable but it contains vitamin I think…...other time in lunch we green leafy vegetables, three times a week" – ND1 |
|  |  | "some time we don't have time cook and some time we do not have food….thats why we are not able to eat diverse food" – ND2 |
|  |  | "I think because of time…...we have lot of work in house so I think we do not have time to prepare many types of food…"- ND9 |
|  | Less availability of food products | "to eat different kind of meat and fish, it requires money. If person is very poor, then they will not get fish and meat to eat. Likewise, if they want to eat peas and beans, they will not because they don't have" – ND2  "I think because of the unavailability of food we are not able eat diverse food "- ND2  "I think because of availability….if we do not have in our home we are not able to eat diverse food" – NDc2 |
|  |  | "Yeah! We should feed green leafy vegetables, fruits, peas and beans to our **children but we do not availability of all food**….....market is also not near us…....also economically we are no strong….that's why we are not able to give food to them" – ND3 |
|  |  | "I think because we don't have all foods in our house…. some time we don't have one things and other time we don't have another things ..that’s the main reason of not consuming diverse food" – NDc3 |
|  |  | "some time we do not have diverse food in home….that's why we are not able to eat diverse vegetables…...."- NDc7 |
|  |  | "we are in village and we are doing agriculture, we don't have many foods available at once. When we do farming some time we have something and sometime we have another things…..if we don't have then we need to buy…..as being a farmer we can eat vegetables, but we cannot eat different kind of diverse food as you said...but we try to eat as much as possible........sometimes economic condition is also important factor" – ND4 |
|  |  | "I don't know much, but we in village we do not have much food like urban areas…." – ND7 |
|  |  | "I think because we don't have diverse food in home, and we cannot buy all things that why we cannot have diverse food"- NDc9 |
|  | Less production | "we eat what we produce…....for other foods we need extra effort….if there was irrigation then we could have good production…. I think that's why people despite of knowledge were not able to provide diverse food"- ND5 |
|  |  | "we are not able to eat diverse food because of the less production, some time we have economic problem…and some time if we have we feel lazy to cook different type of food "- ND6 |
|  | No near market | "Yeah! We should feed green leafy vegetables, fruits, peas and beans to our children but we do not availability of all food…....**.market is also not near us**…....also economically we are no strong….that's why we are not able to give food to them"- ND3 |
|  |  | "we live in rural hills, we do not have access when we want…...some time we get meat twice a week and some time not even for single day"- ND7 |
|  | No strong believe on eating diverse food | "some time people do not have strong belief not consuming diverse food groups…...that may result in not consuming consumption of diverse foods"- ND3 |
|  | Less willingness | "In market either you find same vegetables, or if you find different vegetables but you have vegetable at home then there is less willingness to buy" – ND4  "I think of preference…we do not eat diverse food" – NDc4 |
|  | Laziness | "we are not able to eat diverse food because of the less production, some time we have economic problem…and some time if we have we feel lazy to cook different type of food " |
|  | Don’t want to spend much | "some people might want to save their production or economy, that might be the reason of not consuming diverse food"- ND5 |
|  | No money | "we can't eat diverse food when we do not have money and some time when we are sick" – NDc6 |
|  |  | "I think because of poor economy some people might not get diverse food"- NDc8 |
|  | No idea how to eat | "some people don't know how to eat, even they have wealth….some people eat even by buying…...if chlidren ask they should be provided even by buying …" – ND8 |
