# Supplementary material for: Identifying positive and negative deviants and factors associated with healthy dietary practices among young schoolchildren in Nepal: a mixed methods study
Source: BMC Nutr. 2023 Mar 8;9:42. doi: 10.1186/s40795-023-00700-5 (PMC9993389; doi:10.1186/s40795-023-00700-5)
Supplement: Supplementary file 5 — Additional file 5: [file 40795_2023_700_MOESM5_ESM.docx]

**Supplementary Tables**

**Supplementary Table 1. Characteristics of the schoolchildren, parents, and household, in the control group and treatment group (stratified by deviants) ^±^**

|  |  | **Control group (n=332)** | | | | **Treatment group (n=317)** | | | |
| --- | --- | --- | --- | --- | --- | --- | --- | --- | --- |
| **Variables** | **Category** | **Positive Deviant (n=23)** ^*^  **(%)** | **Non-positive Deviant (n=309) (%)** | **Total**  **n (%)** | **p-value** | **Negative deviants (n=73) (%)** | **Non-negative Deviants (n=244 (%)** | **Total**  **n (%)** | **p-value** |
| Sex | Boy | 9 (39.1) | 137 (44.3) | 146 (44.0) | 0.789 | 35 (47.9) | 108 (44.3) | 143 (45.1) | 0.674 |
|  | Girl | 14 (60.9) | 172 (55.7) | 186 (56.0) |  | 38 (52.1) | 136 (55.7) | 174 (54.9) |  |
| Ethnicity | Brahaman/  Chhetri | 4 (17.3) | 113 (36.6) | 117 (35.2) | 0.149 | 21 (28.8) | 72 (29.5) | 93 (29.3) | 0.416 |
|  | Dalits | 3 (13.04) | 30 (9.7) | 33 (9.9) |  | 3 (4.1) | 21 (8.6) | 24 (7.6) |  |
|  | Adivasi/  Janajati | 16 (69.6) | 166 (53.72) | 182 (54.8) |  | 49 (67.1) | 151(61.9) | 200 (63.1) |  |
| Parents’ literacy | Illiterate | 12 (52.2) | 174 (56.3) | 186 (56.0) | 0.867 | 36 (49.3) | 148 (60.7) | 184 (58) | 0.112 |
|  | Literate^†^ | 11 (47.8) | 135 (43.7) | 146 (44.0) |  | 37 (50.7) | 96 (39.3) | 133 (42) |  |
| Parents’ occupation | Farmer | 18 (78.3) | 241 (78.0) | 259 (78) | 1 | 58 (79.5) | 203 (83.2) | 261(82.3) | 0.575 |
|  | Other than farmer | 5 (21.7) | 68 (22.0) | 73 (22.0) |  | 15 (20.5) | 41 (16.8) | 56(17.7) |  |
| Grandmother in household | Yes | 6 (26.1) | 66 (21.4) | 72 (21.7) | 0.788 | 26 (35.6) | 58 (23.8) | 84 (26.5) | 0.063 |
|  | No | 17 (73.9) | 243 (78.6) | 260 (78.3) |  | 47 (64.4) | 186 (76.2) | 233(73.5) |  |
| Decision-maker for what to cook | Mother | 19 (82.6) | 269 (87.1) | 288 (86.7) | 0.525^#^ | 65 (89.0) | 223 (91.4) | 288 (90.9) | 0.704 |
|  | Other family member | 4 (17.4) | 40 (12.9) | 44 (13.3) |  | 8 (11.0) | 21 (8.6) | 29 (9.1) |  |
| Produced vegetables in home garden | Yes | 18 (78.3) | 271 (87.7) | 289 (87) | 0.198^#^ | 72 (98.6) | 237 (97.1) | 309 (97.5) | 0.687^#^ |
|  | No | 5 (21.7) | 38 (12.3) | 43 (13.0) |  | 1 (1.4) | 7 (2.9) | 8 (2.5) |  |
| Responsible for managing home garden**^‡^** | Mother | 17 (94.4) | 235 (86.7) | 252 (87.2) | 0.486^#^ | 62 (86.1) | 207 (87.3) | 269 (87.1) | 0.943 |
|  | Other family member | 1 (5.6) | 36 (13.3) | 37 (12.8) |  | 10 (13.9) | 30 (12.7) | 40 (12.9) |  |
| Father helping in home garden**^‡^** | Yes | 12 (66.7) | 146 (53.9) | 158 (54.7) | 0.417 | 36 (50.0) | 110 (46.4) | 146 (47.2) | 0.690 |
|  | No | 6 (33.3) | 125 (46.1) | 131 (45.3) |  | 36 (50.0) | 127 (53.6) | 163 (52.8) |  |
| Children assisting in home garden**^‡^** | Yes | 4 (22.2) | 58 (21.4) | 62 (21.5) | 1^#^ | 15 (20.8) | 74 (31.2) | 89 (28.8) | 0.120 |
|  | No | 14 (77.8) | 213 (78.6) | 227 (78.5) |  | 57 (79.2) | 163 (68.8) | 220 (71.2) |  |
| Money received for snacks | Yes | 8 (34.8) | 104 (33.7) | 112 (33.7) | 0.887 | 20 (27.4) | 51 (20.9) | 71 (22.4) | 0.314 |
|  | No | 15 (65.2) | 205 (66.3) | 220 (66.3) |  | 53 (72.6) | 193 (79.1) | 244 (77.6) |  |

^±^ A base package of R-software was used to perform chi-square test (Pearson’s) (χ2); statistical significance < 0.05.

^†^ Literate: those who can read and write; **^‡^**total number of observations is 289 and 307 in the control and treatment group, respectively; ^*^ total number of PDs for variables (**^‡^**) is 18;

**Supplementary Table2. Food consumption in control groups positive deviants (n = 23) and non-positive deviants (n = 309)**

| **Food groups** | **Category** | **Positive deviants**  **n=(23) (%)** | **Non-positive deviants**  **n=(309) (%)** | **Odds Ratio**  **(95% CI)** |
| --- | --- | --- | --- | --- |
| Grains, roots and tubers | Consumed | 23 (100) | 309 (100) | - |
|  | Not consumed | 0 (0) | 0 (0) |  |
| Legumes and nuts | Consumed | 22 (95.7) | 222 (71.8) | 8.62*  (1.14 - 64.95) |
|  | Not consumed | 1 (4.3) | 87 (28.2) |  |
| Dairy | Consumed | 8 (34.8) | 30 (9.7) | 4.96***  (1.94 - 12.66) |
|  | Not consumed | 15 (65.2) | 279 (90.3) |  |
| Eggs | Consumed | 6 (26.1) | 18 (5.8) | 5.71***  (2.01 - 16.23) |
|  | Not consumed | 17 (73.9) | 291(94.2) |  |
| Flesh Foods | Consumed | 14 (60.9) | 105 (34.0) | 3.02*  (1.27 - 7.21) |
|  | Not consumed | 9 (39.1) | 204 (66.0) |  |
| Vitamin-A rich fruits and vegetables | Consumed | 11 (47.8) | 95 (30.7) | 2.06  (0.88 - 4.85) |
|  | Not consumed | 12 (52.2) | 214 (69.3) |  |
| Other fruits and vegetables | Consumed | 13 (56.5) | 181 (58.6) | 0.92  (0.39 - 2.16) |
|  | Not consumed | 10 (43.5) | 128 (41.4) |  |

**Note:** *epiR* package of R-software was used to perform chi-square test (Mantel-Haenszel) and odds ratio (Wald). CI: confidence Interval, significance * < 0.05, ** < 0.01, *** < 0.001

**Supplementary Table 3. Food consumption by negative deviants (n=73) and non-negative deviants (n = 244) in the intervention group**

| **Food groups** | **Category** | **Negative deviants**  **n=(73) (%)** | **Non-negative deviants**  **n=(244) (%)** | **Odds Ratio**  **(95% CI)** |
| --- | --- | --- | --- | --- |
| Grains, roots and tubers | Consumed | 73 (100%) | 244 (100%) | - |
|  | Not consumed | 0 | 0 |  |
| Legumes and nuts | Consumed | 44 (60.3) | 184 (75.4) | 0.49*  (0.28 - 0.86) |
|  | Not consumed | 29 (39.7) | 60 (24.6) |  |
| Dairy products | Consumed | 2 (2.7) | 34 (13.9) | 0.17*  (0.04 - 0.74) |
|  | Not consumed | 71 (97.3) | 210 (86.1) |  |
| Eggs | Consumed | 3 (4.1) | 13(5.3) | 0.76  (0.21 - 2.75) |
|  | Not consumed | 70 (95.9) | 231 (94.7) |  |
| Flesh foods | Consumed | 33 (45.2) | 106 (43.4) | 1.07  (0.63 - 1.82) |
|  | Not consumed | 40 (54.8) | 138 (56.6) |  |
| Vitamin-A rich fruits and vegetables | Consumed | 22 (30.1) | 90 (36.9) | 0.74  (0.42 - 1.30) |
|  | Not consumed | 51 (69.9) | 154 (63.1) |  |
| Other fruits and vegetables | Consumed | 53 (72.6) | 143 (58.6) | 1.87*  (1.05 - 3.32) |
|  | Not consumed | 20 (27.4) | 101 (41.4) |  |

**Note:** *epiR* package of R-software was used to perform (Mantel-Haenszel) chi-square test and (Wald) odds ratio; CI: confidence Interval; significance: * < 0.05, ** < 0.01
